# Bullous keratopathy associated with a shallow anterior chamber: An anatomical risk phenotype for corneal endothelial decompensation

**DOI:** 10.1371/journal.pone.0353272

**Published:** 2026-07-07

**Authors:** Masato Takeda, Yuki Mizuki, Ami Igarashi, Toshiki Shimizu, Naoki Okumura, Nobuhisa Mizuki, Satoru Yamagami, Takahiko Hayashi

**Affiliations:** 1 Department of Ophthalmology, Yokohama Minami Kyosai Hospital, Yokohama, Japan; 2 Department of Ophthalmology and Visual Science, Yokohama City University Graduate School of Medicine, Yokohama, Japan; 3 Department of Ophthalmology, Department of Visual Sciences, Nihon University School of Medicine, Tokyo, Japan; 4 Kikuna Yuda Eye Clinic, Yokohama, Japan; 5 Department of Biomedical Engineering, Faculty of Life and Medical Sciences, Doshisha University, Kyoto, Japan; University at Buffalo Jacobs School of Medicine and Biomedical Sciences: University at Buffalo School of Medicine and Biomedical Sciences, UNITED STATES OF AMERICA

## Abstract

**Purpose:**

To characterize bullous keratopathy (BK) associated with a shallow anterior chamber (AC) and to examine whether a shallow AC itself may be associated with corneal endothelial decompensation, even in the absence of acute primary angle closure (APAC) or argon laser iridotomy (ALI).

**Methods:**

This multicenter retrospective observational study included 96 eyes that underwent Descemet membrane endothelial keratoplasty (DMEK) for endothelial dysfunction between 2015 and 2025 with ≥12 months of follow-up. The primary comparative analyses compared eyes with Fuchs endothelial corneal dystrophy (FECD) and eyes with endothelial dysfunction associated with a shallow AC. The shallow anterior chamber phenotype group comprised eyes with a documented history of APAC or ALI (PACD/ALI) and those without a documented history of either condition (shallow-AC). Anterior chamber depth (ACD), axial length, corneal thickness, endothelial parameters, and postoperative outcomes were compared between groups.

**Results:**

Pre-cataract ACD was significantly shallower in the shallow anterior chamber phenotype group than in the FECD group (1.93 ± 0.40 mm vs. 2.28 ± 0.27 mm, P < 0.001), and this relative difference persisted after cataract surgery. Within the shallow anterior chamber phenotype group, 10 eyes were classified as shallow-AC and had no documented history of APAC or ALI. Five-year graft survival after DMEK exceeded 90% and did not differ significantly between the shallow anterior chamber phenotype and FECD groups, nor between PACD/ALI and shallow-AC subgroups.

**Conclusions:**

A shallow anterior chamber may be associated with corneal endothelial decompensation, even in the absence of APAC or ALI. With appropriate perioperative management, DMEK may provide favorable long-term outcomes in these eyes.

## Introduction

Corneal endothelial dysfunction is a major indication for endothelial keratoplasty, although the underlying etiologies vary according to geographic region and patient demographics [1–3]. In Western countries, Fuchs endothelial corneal dystrophy (FECD) accounts for the majority of cases, whereas in East Asian populations, secondary forms of bullous keratopathy (BK), including pseudophakic and angle-closure–related endothelial failure, represent a larger proportion of surgical indications [3–5]. Despite advances in surgical techniques, the mechanisms underlying endothelial decompensation in non-FECD eyes remain incompletely understood.

Eyes with primary angle closure (PAC) or a history of acute primary angle closure (APAC) are well recognized to be at increased risk for corneal endothelial cell loss [6]. In particular, endothelial injury following argon laser iridotomy (ALI) has been extensively documented, and ALI-related BK has emerged as a clinically important entity, especially in Asian countries [7,8]. These observations have traditionally framed endothelial failure in PAC eyes as a consequence of discrete injurious events, such as acute intraocular pressure elevation or laser-induced alterations in aqueous humor flow dynamics.

However, accumulating clinical evidence suggests that anatomical factors may play a more fundamental and chronic role in endothelial stress. A shallow anterior chamber (AC) and a crowded anterior segment configuration are characteristic features of the angle-closure spectrum and may contribute to surgical difficulty and postoperative anterior segment changes following endothelial keratoplasty [9,10]. These findings raise the possibility that sustained anatomical conditions, rather than isolated acute events, may contribute to progressive endothelial damage.

The distinction between primary endothelial disease and anatomically mediated endothelial failure is further complicated by recent reports suggesting that eyes with corneal guttae or FECD may have a relatively shallow anterior chamber [11]. This overlap suggests that anterior segment anatomy may modulate endothelial vulnerability across different disease categories and underscores the need to better characterize anatomical risk profiles independent of traditional diagnostic labels.

Despite these observations, it remains unclear whether a shallow anterior chamber alone—without a history of APAC or ALI—is sufficient to predispose eyes to progressive endothelial decompensation and BK. To date, most clinical studies examining BK in the context of angle closure have focused on eyes with documented acute attacks or prior iridotomy, and a distinct phenotype characterized primarily by anatomical shallowing of the anterior chamber has not been clearly delineated [7,8].

From a surgical standpoint, eyes with shallow anterior chambers have historically been considered technically challenging for Descemet membrane endothelial keratoplasty (DMEK), owing to limited intraocular working space, increased vitreous pressure, and a higher risk of graft instability [12]. Nevertheless, advances in perioperative management and surgical technique have improved the feasibility of DMEK in such eyes, and recent studies have demonstrated favorable outcomes in eyes with shallow anterior chambers and angle-closure– related endothelial failure [7,9,12]. Whether the anatomical risk associated with a shallow AC necessarily translates into inferior long-term graft survival, however, remains uncertain.

The primary aim of the present multicenter retrospective study was therefore to characterize bullous keratopathy associated with a shallow anterior chamber as a potential anatomical risk phenotype for corneal endothelial decompensation, with particular emphasis on eyes lacking any history of APAC or ALI. As a secondary objective, we evaluated long-term outcomes after DMEK in these eyes to assess whether favorable graft survival can be achieved in the setting of this anatomical configuration.

## Materials and methods

### Study design and ethics

This multicenter retrospective observational study was approved by the Institutional Review Boards of all participating institutions, including Nihon University Itabashi Hospital (approval number: RK-230411-8). Owing to the retrospective nature of the study, the requirement for written informed consent was waived, and an opt-out policy was implemented in accordance with institutional regulations.

The study population comprised eyes that underwent DMEK at Yokohama Minami Kyosai Hospital and Kikuna Yuda Eye Clinic between April 1, 2015, and March 31, 2025. Clinical data were accessed and analyzed only by investigators who were formally approved members or collaborators under each institutional review board between May 1, 2025, and August 31, 2025. Data sharing across institutions was conducted in a fully anonymized manner, and no personally identifiable information was accessible to investigators outside the originating institution.

### Patients and data collection

This study included 145 consecutive eyes with corneal endothelial decompensation that underwent DMEK at Yokohama Minami Kyosai Hospital and Kikuna Yuda Eye Clinic between April 2015 and March 2025. Only eyes with at least 12 months of postoperative or follow-up data were eligible for inclusion. Patients younger than 20 years, those with insufficient clinical or imaging data, and those without at least 12 months of follow-up were excluded.

Demographic and clinical information was collected from medical records, including age, sex, systemic conditions, laterality, lens status, and history of intraocular surgery such as cataract surgery and laser iridotomy. The underlying etiology of endothelial decompensation was recorded for each case. The diagnosis of BK was based on the presence of persistent stromal and epithelial edema with bullae and a clinical impression of endothelial failure. For the purposes of this study, the BK category comprised eyes with primary angle-closure disease (PACD) with prior argon laser iridotomy (PACD/ALI), shallow anterior chamber (shallow-AC), pseudoexfoliation (PEX), aphakic bullous keratopathy (ABK), and pseudophakic bullous keratopathy (PBK). FECD was defined by the presence of characteristic guttae with or without corneal edema in the absence of angle-closure features. In cases with advanced corneal edema that obscured endothelial details, the diagnosis was made based on longitudinal documentation and clinical findings of the fellow eye.

The PACD/ALI subgroup was defined as eyes with endothelial decompensation and a documented history of ALI performed for a clinically diagnosed PACD-spectrum condition, mainly primary angle closure or primary angle-closure glaucoma, according to conventional criteria [13]. The diagnosis of PACD was based on medical records documenting gonioscopic angle closure and/or a clinical diagnosis by glaucoma or corneal specialists. Because this was a retrospective study, standardized gonioscopic grading and the extent of peripheral anterior synechiae were not consistently available and were therefore not included as analytic variables. The shallow-AC subgroup was defined as eyes with endothelial decompensation and a clinically shallow anterior chamber configuration, but without documented APAC or ALI history. A clinically shallow anterior chamber was defined as Van Herick grade 2 or lower on slit-lamp examination [14]. This classification was used to define a shallow anterior chamber phenotype and was not intended to represent a strict diagnosis of PACD based on uniform gonioscopic criteria.

The study included eyes that underwent DMEK for endothelial dysfunction of various etiologies. Among these, the primary comparative analyses were performed between eyes with FECD and eyes with endothelial dysfunction associated with a shallow anterior chamber phenotype. The shallow anterior chamber phenotype included the PACD/ALI subgroup and the shallow-AC subgroup. Subgroup analyses compared the PACD/ALI subgroup and the shallow-AC subgroup. Perioperative variables included the type of surgery performed, donor age, the occurrence and number of rebubbling procedures, the number of additional air injections, and postoperative complications such as rejection or late endothelial failure. Additionally, pre-DMEK intraocular pressure (IOP) and the use of pressure-lowering medication were recorded.

### Ophthalmic examinations

Clinical parameters recorded before DMEK included best-corrected visual acuity (BCVA), central corneal thickness (CCT), intraocular pressure (IOP), the number of IOP-lowering medications, axial length, and preoperative donor corneal ECD. Preoperative VA and preoperative CCT referred to measurements obtained before DMEK. Preoperative ECD referred to the endothelial cell density of the donor corneal graft and not the recipient eye before DMEK. ACD was evaluated separately as pre-cataract surgery ACD and post-cataract surgery ACD. Post-cataract surgery ACD was measured at the pre-DMEK assessment. BCVA was measured using standard decimal visual acuity charts and converted to logarithm of the minimum angle of resolution (logMAR) values for analysis. CCT and ACD were measured using anterior segment optical coherence tomography (CASIA; Tomey, Nagoya, Japan), and axial length was measured by optical biometry (OA-2000; Tomey, Nagoya, Japan). Specular microscopy was performed using a noncontact specular microscope (Konan Medical, Hyogo, Japan). On specular microscopy, dark spots were defined as focal dark hyporeflective areas within the endothelial mosaic, occasionally accompanied by a central white reflex, corresponding to previously described specular microscopic appearances of corneal guttae in FECD [15]. Dark spots alone were not used as independent diagnostic criteria for FECD. AS-OCT was used to measure ACD and CCT, but angle parameters, including the trabecular iris angle, were not uniformly measured and were not used for group classification or statistical analyses.

### Surgical procedures

All included eyes underwent DMEK, which was performed using standardized techniques. In eyes with a shallow anterior chamber, intraoperative adaptations, such as chamber pressure control and careful graft unfolding, were used at the surgeon’s discretion. When necessary, an anterior chamber maintainer or low-viscosity viscoelastic was applied during graft insertion, followed by meticulous removal to avoid interference with graft attachment.

Postoperative management consisted of topical antibiotics and corticosteroids administered with a tapered regimen, along with supine positioning during the early postoperative period, in accordance with the institutional standard protocol.

### Statistical analysis

Demographic and clinical variables were summarized descriptively, with continuous variables presented as mean ± SD and median [range], and categorical variables as n [%]. To evaluate potential selection bias, cases were stratified by the availability of pre-cataract surgery ACD. Pre-cataract surgery ACD was analyzed on an available-case basis, and no imputation was performed for missing values. The 35 eyes with missing pre-cataract surgery ACD had already undergone cataract surgery at outside institutions and were pseudophakic at both the initial visit and pre-keratoplasty assessment. The association between pre-cataract surgery and post-cataract surgery ACD was assessed with Pearson’s correlation and visualized using a scatterplot overlaid with kernel-density contours. Determinants of ACD were examined using univariate linear regression models run separately for pre-cataract surgery (n = 61) and post-cataract surgery (n = 96) ACD. Effect sizes for continuous predictors are expressed per 1-SD increase, and those for categorical predictors relative to appropriate reference categories. Graft survival was analyzed using Kaplan–Meier methods and compared with the log-rank test after dichotomizing ACD at the cohort medians (pre-cataract surgery, 2.14 mm; post-cataract surgery, 3.85 mm). Group comparisons were performed using the Wilcoxon rank-sum test for continuous or ordinal variables and Fisher’s exact test for categorical variables. Main comparisons included the shallow anterior chamber phenotype group versus FECD and the PACD/ALI subgroup versus the shallow-AC subgroup. Statistical significance was defined as two-sided P < 0.05, without adjustment for multiple comparisons. Analyses were performed using R version 4.5.1 (R Foundation for Statistical Computing, Vienna, Austria).

## Results

### Patient characteristics

**Fig 1:**
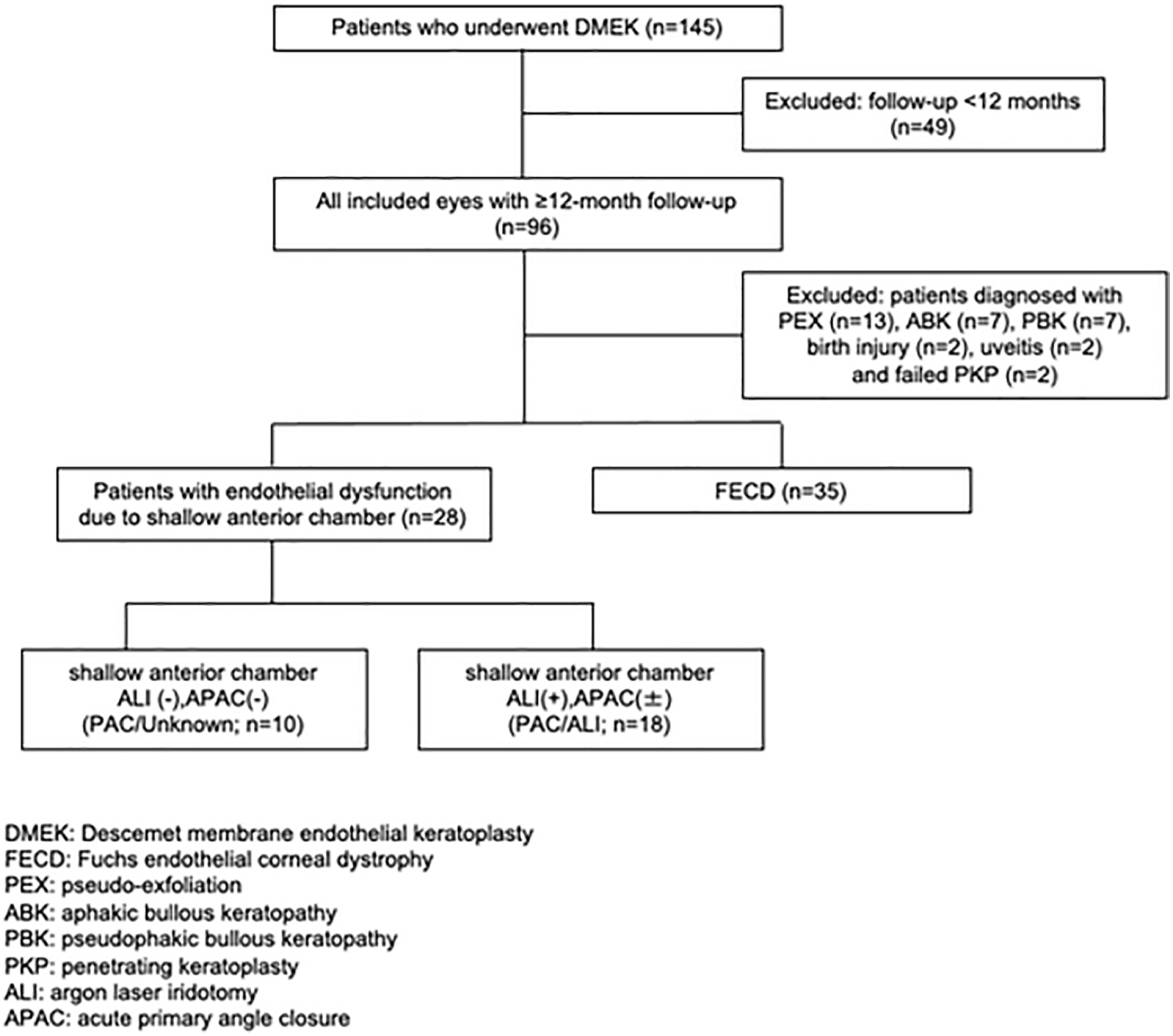
Summary of the study flow. We identified 145 eyes that underwent DMEK. After excluding 49 eyes with <12 months of follow-up, 96 eyes remained. Of these, the etiologies were FECD (n = 35), the shallow anterior chamber phenotype group (n = 28), PEX (n = 13), aphakic bullous keratopathy (ABK; n = 7), pseudophakic bullous keratopathy (PBK; n = 7), birth injury (n = 2), uveitis (n = 2), and failed penetrating keratoplasty (n = 2). The shallow anterior chamber phenotype group was further subdivided into PACD/ALI (n = 18) and shallow-AC (n = 10). DMEK = Descemet membrane endothelial keratoplasty; FECD = Fuchs endothelial corneal dystrophy; PEX = pseudoexfoliation; ABK = aphakic bullous keratopathy; PBK = pseudophakic bullous keratopathy, PKP = penetrating keratoplasty, PACD = primary angle-closure disease, ALI = argon laser iridotomy; APAC = acute primary angle closure.

Fig 1 summarizes the study flow. We identified 145 eyes that underwent DMEK. After excluding 49 eyes with <12 months of follow-up, 96 eyes were included in the final analysis.

**Fig 2:**
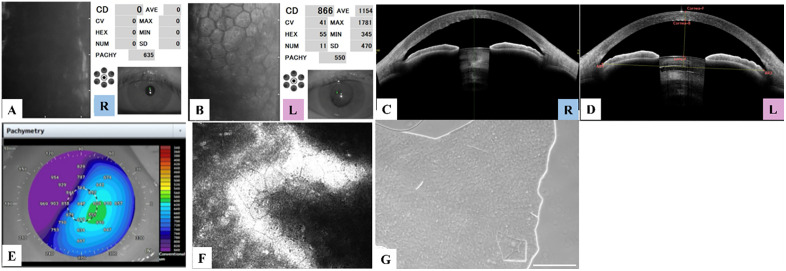
Evaluation of corneal endothelium in bullous keratopathy associated with a shallow anterior chamber without documented history of APAC or laser iridotomy. (a) Specular microscopy in the affected eye (upper panel), showing a dark area due to severe corneal endothelial cell injury. (b) Image of the fellow eye, showing no corneal guttae, from an illustrative case in the shallow-AC subgroup, defined as eyes with a clinically shallow anterior chamber configuration but without documented APAC or ALI history. (c) Anterior segment optical coherence tomography (CASIA; Tomey, Nagoya, Japan) (horizontal temporal-to-nasal scan) in the affected eye, showing thickening and irregular posterior surface due to corneal endothelial cell damage. (d) The fellow eye of this shallow-AC case shows no corneal edema or folds in the Descemet’s membrane. (e) Pachymetry measured via anterior segment optical coherence tomography demonstrated asymmetric thickening with a central corneal thickness of approximately 740 µm and maximal values near 950 µm. (f) In vivo confocal microscopy demonstrated endothelial dropout with fibrous/reflective deposits rather than a diffuse, guttate-dominant pattern in a case from the shallow-AC subgroup. (g) Transmission electron micrograph of Descemet’s membrane from a shallow-AC case. The posterior surface appears relatively smooth without nodular guttae. **Abbreviations:** APAC = acute primary angle closure; shallow-AC = shallow anterior chamber.

Of these, the etiologies were FECD (n = 35), the shallow anterior chamber phenotype group (n = 28), PEX (n = 13), aphakic BK (n = 7), pseudophakic BK (n = 7), birth injury (n = 2), uveitis (n = 2), and failed penetrating keratoplasty (n = 2). The shallow anterior chamber phenotype group was further subdivided into PACD/ALI (n = 18) and shallow-AC (n = 10). Table 1 summarizes patient demographics and clinical characteristics, and Table 2 shows the breakdown of BK cases. Overall, 96 eyes from 78 patients were included in the study (mean age, 74.8 ± 8.2 years), and 69 eyes (71.9%) were female. Pre-cataract surgery ACD measurements were available in 61 eyes (63.5%). As shown in Table 2, missing pre-cataract surgery ACD data were not equally distributed across BK subgroups; all ABK and PBK eyes lacked pre-cataract surgery ACD measurements, whereas all shallow-AC eyes had available measurements. Overall, BK accounted for the largest proportion of cases (57.3%), followed by FECD (36.5%). Among eyes with pre-cataract surgery ACD measurements, mean ACD increased from 2.18 ± 0.49 mm before cataract surgery to 3.86 ± 0.37 mm at the post-cataract surgery, pre-DMEK assessment. As an illustrative case from the shallow-AC subgroup, Fig 2 presents multimodal findings in the affected and fellow eyes, including specular microscopy, corneal tomographic findings, in vivo confocal microscopy, and a representative specimen of diseased Descemet membrane prepared for electron microscopy as previously described [12] and imaged with a transmission electron microscope (EM 906E; Carl Zeiss Microscopy, Oberkochen, Germany). All data used in this analysis are provided in the dataset (S1 Dataset in S1 File).

**Table 1:** Patient demographics and clinical characteristics.

Item [unit]	Statistics Category	Overall (N = 96)	Pre-cataract surgery ACD available (N = 61)	Pre-cataract surgery ACD missing (N = 35)
Age [years]	Mean (SD), N	74.8 (8.2), 96	74.7 (7.8), 61	74.8 (9.0), 35
	Median [Range]	75.0 [54, 89]	75.0 [54, 85]	74.0 [55, 89]
Sex	[1] Male	27 (28.1%)	17 (27.9%)	10 (28.6%)
	[2] Female	69 (71.9%)	44 (72.1%)	25 (71.4%)
Disease	[1] BK	55 (57.3%)	32 (52.5%)	23 (65.7%)
	[2] FECD	35 (36.5%)	24 (39.3%)	11 (31.4%)
	[3] Failed PKP	2 (2.1%)	2 (3.3%)	0 (0.0%)
	[4] Birth injury	2 (2.1%)	2 (3.3%)	0 (0.0%)
	[5] Uveitis	2 (2.1%)	1 (1.6%)	1 (2.9%)
Pre-cataract surgery ACD [mm]	Mean (SD), N	2.18 (0.49), 61	2.18 (0.49), 61	–
	Median [Range]	2.14 [1.3, 3.4]	2.14 [1.3, 3.4]	–
Post-cataract surgery ACD [mm]	Mean (SD), N	3.85 (0.46), 96	3.86 (0.37), 61	3.83 (0.60), 35
	Median [Range]	3.85 [2.8, 5.3]	3.84 [3.2, 4.6]	3.89 [2.8, 5.3]
Laser iridotomy	[1] No	78 (81.3%)	48 (78.7%)	30 (85.7%)
	[2] Yes	18 (18.7%)	13 (21.3%)	5 (14.3%)
Acute primary angle closure	[1] No	88 (91.7%)	57 (91.8%)	31 (88.6%)
	[2] Yes	8 (8.3%)	4 (8.2%)	4 (11.4%)
Rebubbling	[1] No	67 (69.8%)	40 (65.6%)	27 (77.1%)
	[2] Yes	29 (30.2%)	21 (34.4%)	8 (22.9%)
Pre-DMEK IOP [mmHg]	Mean (SD), N	12.5 (3.7), 96	12.2 (3.8), 61	12.9 (3.4), 35
	Median [Range]	12.0 [6 22]	12.0 [6 21]	12.0 [6 22]
Number of IOP-lowering medications before DMEK [counts]	[1] 0	84 (87.5%)	55 (90.2%)	29 (82.9%)
	[2] 1	4 (4.2%)	3 (4.9%)	1 (2.9%)
	[3] 2	6 (6.2%)	3 (4.9%)	3 (8.6%)
	[4] 3	2 (2.1%)	0 (0.0%)	2 (5.7%)
Axial length [mm]	Mean (SD), N	23.37 (1.67), 96	23.26 (1.38), 61	23.56 (2.10), 35
	Median [Range]	22.99 [21.0, 31.6]	22.89 [21.2, 27.2]	23.19 [21.0, 31.6]
Pre-DMEK VA [logMAR]	Mean (SD), N	0.746 (0.485), 96	0.725 (0.500), 61	0.785 (0.463), 35
	Median [Range]	0.699 [−0.08, 2.00]	0.523 [−0.00, 2.00]	0.699 [−0.08, 2.00]
Visual acuity at last visit [logMAR]	Mean (SD), N	0.184 (0.345), 96	0.137 (0.294), 61	0.266 (0.410), 35
	Median [Range]	0.097 [−0.08, 2.00]	0.097 [−0.08, 2.00]	0.155 [−0.08, 2.00]
Preoperative donor ECD [cells/mm²]	Mean (SD), N	2655.5 (205.0), 96	2650.9 (217.8), 61	2663.5 (183.1), 35
	Median [Range]	2632.0 [2020, 3313]	2632.0 [2020, 3227]	2632.0 [2404, 3313]
ECD at 12 months [cells/mm²]	Mean (SD), N	1189.9 (479.8), 96	1280.4 (515.3), 61	1032.0 (366.4), 35
	Median [Range]	1062.0 [363, 2519]	1159.0 [363, 2519]	947.0 [465, 2110]
Loss of ECD at 12 months [%]	Mean (SD), N	55.67 (16.88), 96	52.40 (17.99), 61	61.37 (13.12), 35
	Median [Range]	58.75 [13.7, 86.1]	54.90 [13.7, 86.1]	64.51 [16.8, 81.6]
ECD at last visit [cells/mm²]	Mean (SD), N	975.6 (413.4), 92	1050.7 (444.2), 59	841.2 (315.3), 33
	Median [Range]	839.0 [360, 2532]	911.0 [360, 2532]	750.0 [465, 1898]
Pre-DMEK CCT [µm]	Mean (SD), N	685.0 (96.3), 96	693.3 (100.1), 61	670.5 (89.0), 35
	Median [Range]	673.0 [501, 956]	681.0 [501, 937]	656.0 [553, 956]
CCT at last visit [µm]	Mean (SD), N	526.9 (53.9), 96	522.3 (52.0), 61	535.1 (56.8), 35
	Median [Range]	520.0 [412, 767]	515.0 [412, 687]	522.0 [457, 767]
Dark spots on specular microscopy	[1] No	56 (58.3%)	40 (65.6%)	16 (45.7%)
	[2] Yes	40 (41.7%)	21 (34.4%)	19 (54.3%)
CME onset	[1] No	83 (86.5%)	55 (90.2%)	28 (80.0%)
	[2] Yes	13 (13.5%)	6 (9.8%)	7 (20.0%)
Graft failure	[1] No	87 (90.6%)	58 (95.1%)	29 (82.9%)
	[2] Yes	9 (9.4%)	3 (4.9%)	6 (17.1%)
Follow-up days until graft failure	Mean (SD), N	1602.2 (715.1), 96	1687.4 (703.9), 61	1453.5 (720.2), 35
	Median [Range]	1741.5 [315, 3089]	1858.0 [315, 3089]	1562.0 [403, 2948]

Abbreviations: ACD = anterior chamber depth; ALI = argon laser iridotomy; APAC = acute primary angle closure; BK = bullous keratopathy; CCT = central corneal thickness; CME = cystoid macular edema; ECD = endothelial cell density; FECD = Fuchs endothelial corneal dystrophy; IOP = intraocular pressure; logMAR = logarithm of the minimum angle of resolution; PKP = penetrating keratoplasty; SD = standard deviation.

**Table 2:** Breakdown of bullous keratopathy cases.

BK subgroup	Overall BK cases (N = 55)	Pre-cataract surgery ACD available BK cases (N = 32)	Pre-cataract surgery ACD missing BK cases (N = 23)
PACD/ALI	18 (32.7%)	13 (40.6%)	5 (21.7%)
shallow-AC	10 (18.2%)	10 (31.3%)	0 (0.0%)
PEX	13 (23.6%)	9 (28.1%)	4 (17.4%)
ABK	7 (12.7%)	0 (0.0%)	7 (30.4%)
PBK	7 (12.7%)	0 (0.0%)	7 (30.4%)

Abbreviations: ABK = aphakic bullous keratopathy; ALI = argon laser iridotomy; BK = bullous keratopathy; PACD = primary angle-closure disease; shallow-AC = shallow anterior chamber; PBK = pseudophakic bullous keratopathy; PEX = pseudoexfoliation. Percentages were calculated among BK cases.

Additional cataract surgery-related variables were reviewed in the shallow anterior chamber phenotype group (S2 Table in S1 File). Among these 28 eyes, 22 (78.6%) underwent cataract surgery as part of a planned staged surgical approach before DMEK after corneal endothelial decompensation or BK had already been recognized. Cumulative dissipated energy was not available in the medical records. Among eyes with available IOL position data, all IOLs were placed in the bag, and no sulcus IOL or anterior chamber IOL was documented. S2 Table in S1 File summarizes the interval from ALI to cataract surgery and recipient ECD before cataract surgery. Clinical findings suggestive of viral endotheliitis were noted in one eye, and aqueous humor testing, when performed, was negative.

### Factors associated with ACD

S3 Fig in S1 File depicts a scatter plot with density contours showing pre- versus post-cataract surgery ACD values. Lower pre-cataract surgery ACD values tended to have relatively lower post-cataract surgery ACD.

Table 3 presents the results of univariate regression for pre-cataract surgery ACD. Female sex was associated with lower ACD than male sex (–0.34 mm, 95% CI: –0.61 to –0.07, P = 0.014). FECD was associated with higher ACD than BK (+0.30 mm, 95% CI: 0.09 to 0.52, P < 0.001). ALI (–0.59 mm, 95% CI: –0.86 to –0.32, P < 0.001) and APAC (–0.70 mm, 95% CI: –1.18 to –0.22, P = 0.005) were associated with lower ACD, whereas longer axial length was associated with higher ACD (0.36 mm per SD, 95% CI: 0.24 to 0.49, P < 0.001).

**Table 3:** Univariate analysis of pre-cataract surgery ACD [mm].

Explanatory variable	Category Unit	Pre-cataract surgery ACD [mm] Mean (SD), N	Coefficient		P value
			Estimate	95% C.I.	
Age	1SD increase	2.18 (0.49), 61	−0.13	[-0.26, 0.01]	0.059
Sex	[1] Male	2.43 (0.49), 17	Reference	–	0.014
	[2] Female	2.09 (0.46), 44	−0.34	[-0.61, -0.07]	
Disease	[1] BK	1.97 (0.47), 32	Reference	–	< 0.001
	[2] FECD	2.28 (0.27), 24	0.30	[0.09, 0.52]	
	[3] Failed PKP	3.17 (0.21), 2	1.20	[0.62, 1.77]	
	[4] Birth injury	3.12 (0.39), 2	1.15	[0.57, 1.72]	
	[5] Uveitis	2.83 (-), 1	0.86	[0.06, 1.66]	
Breakdown of BK	[1] PACD/ALI	1.72 (0.24), 13	Reference	–	0.032
	[2] shallow-AC	2.20 (0.43), 10	0.48	[0.11, 0.85]	
	[3] PEX	2.09 (0.62), 9	0.36	[-0.02, 0.75]	
Laser iridotomy	[1] No	2.31 (0.47), 48	Reference	–	< 0.001
	[2] Yes	1.72 (0.24), 13	−0.59	[-0.86, -0.32]	
Acute primary angle closure	[1] No	2.23 (0.47), 57	Reference	–	0.005
	[2] Yes	1.53 (0.22), 4	−0.70	[-1.18, -0.22]	
Rebubbling	[1] No	2.24 (0.53), 40	Reference	–	0.243
	[2] Yes	2.08 (0.41), 21	−0.16	[-0.42, 0.11]	
Pre-DMEK IOP [mmHg]	1SD increase	2.18 (0.49), 61	−0.02	[-0.14, 0.10]	0.763
Number of IOP-lowering medications before DMEK [counts]	[1] 0	2.19 (0.50), 55	Reference	–	0.872
	[2] 1	2.22 (0.52), 3	0.04	[-0.56, 0.63]	
	[3] 2	2.04 (0.57), 3	−0.15	[-0.74, 0.44]	
Axial length [mm]	1SD increase	2.18 (0.49), 61	0.36	[0.24, 0.49]	< 0.001
Pre-DMEK VA [logMAR]	1SD increase	2.18 (0.49), 61	−0.08	[-0.20, 0.04]	0.196
VA at last visit [logMAR]	1SD increase	2.18 (0.49), 61	0.02	[-0.13, 0.17]	0.815
Preoperative donor ECD [cells/mm²]	1SD increase	2.18 (0.49), 61	0.09	[-0.03, 0.21]	0.120
ECD at 12 months [cells/mm²]	1SD increase	2.18 (0.49), 61	0.00	[-0.12, 0.12]	0.945
Loss of ECD at 12 months [%]	1SD increase	2.18 (0.49), 61	0.02	[-0.10, 0.14]	0.782
ECD at last visit [cells/mm²]	1SD increase	2.18 (0.50), 59	0.06	[-0.07, 0.18]	0.357
Pre-DMEK CCT [µm]	1SD increase	2.18 (0.49), 61	−0.09	[-0.22, 0.03]	0.121
CCT at last visit [µm]	1SD increase	2.18 (0.49), 61	−0.03	[-0.16, 0.10]	0.648
Dark spots on specular microscopy	[1] No	2.16 (0.52), 40	Reference	–	0.544
	[2] Yes	2.24 (0.43), 21	0.08	[-0.19, 0.35]	
CME onset	[1] No	2.18 (0.49), 55	Reference	–	0.925
	[2] Yes	2.20 (0.51), 6	0.02	[-0.41, 0.45]	
Graft failure	[1] No	2.19 (0.50), 58	Reference	–	0.847
	[2] Yes	2.13 (0.27), 3	−0.06	[-0.64, 0.53]	

Standard deviations (SD): Age = 8.2 years, Pre-DMEK IOP = 3.7 mmHg, Axial length = 1.67 mm, Pre-DMEK VA = 0.485 logMAR, VA at last visit = 0.345 logMAR, Preoperative donor ECD = 205.0 cells/mm², ECD at 12 months = 479.8 cells/mm², Loss of ECD at 12 months = 16.88%, ECD at last visit = 413.4 cells/mm², Pre-DMEK CCT = 96.3 µm, CCT at last visit = 53.9 µm.

Abbreviations: ACD = anterior chamber depth; ALI = argon laser iridotomy; VA = visual acuity; BK = bullous keratopathy; CCT = central corneal thickness; CI = confidence interval; CME = cystoid macular edema; ECD = endothelial cell density; FECD = Fuchs endothelial corneal dystrophy; IOP = intraocular pressure; logMAR = logarithm of the minimum angle of resolution; PACD = primary angle closure disease; shallow-AC = shallow anterior chamber; PEX = pseudoexfoliation; PKP = penetrating keratoplasty; SD = standard deviation.

Table 4 summarizes the regression results for post-cataract surgery ACD. FECD was associated with higher ACD than BK (+0.40 mm, 95% CI: 0.21 to 0.58, P = 0.001). Among BK cases, PEX was associated with higher ACD than PACD/ALI (+0.36 mm, 95% CI: 0.07 to 0.65, P = 0.030). ALI (–0.30 mm, 95% CI: –0.53 to –0.06, P = 0.013), APAC (–0.39 mm, 95% CI: –0.72 to –0.05, P = 0.024), and lower final CCT (–0.10 mm per SD, 95% CI: –0.20 to –0.01, P = 0.027) were associated with lower post-cataract surgery ACD. In these analyses, BK comprised PACD/ALI, shallow-AC, PEX, ABK, and PBK.

**Table 4:** Univariate analysis of post-cataract surgery ACD [mm].

Explanatory variable	Category Unit	Post-cataract surgery ACD [mm] Mean (SD), N	Coefficient		P value
			Estimate	95% C.I.	
Age	1SD increase	3.85 (0.46), 96	0.03	[-0.06, 0.13]	0.472
Sex	[1] Male	3.97 (0.39), 27	Reference	–	0.122
	[2] Female	3.80 (0.48), 69	−0.16	[-0.37, 0.04]	
Disease	[1] BK	3.68 (0.42), 55	Reference	–	0.001
	[2] FECD	4.08 (0.44), 35	0.40	[0.21, 0.58]	
	[3] Failed PKP	3.98 (0.19), 2	0.29	[-0.32, 0.91]	
	[4] Birth injury	4.21 (0.62), 2	0.52	[-0.09, 1.14]	
	[5] Uveitis	3.83 (0.23), 2	0.15	[-0.46, 0.76]	
Breakdown of BK	[1] PACD/ALI	3.61 (0.29), 18	Reference	–	0.030
	[2] shallow-AC	3.75 (0.38), 10	0.15	[-0.17, 0.46]	
	[3] PEX	3.97 (0.40), 13	0.36	[0.07, 0.65]	
	[4] ABK	3.43 (0.39), 7	−0.17	[-0.53, 0.18]	
	[5] PBK	3.51 (0.61), 7	−0.10	[-0.46, 0.25]	
Laser iridotomy	[1] No	3.90 (0.48), 78	Reference	–	0.013
	[2] Yes	3.61 (0.29), 18	−0.30	[-0.53, -0.06]	
Acute primary angle closure	[1] No	3.88 (0.46), 88	Reference	–	0.024
	[2] Yes	3.50 (0.31), 8	−0.39	[-0.72, -0.05]	
Rebubbling	[1] No	3.81 (0.48), 67	Reference	–	0.268
	[2] Yes	3.93 (0.41), 29	0.11	[-0.09, 0.32]	
Pre-DMEK IOP [mmHg]	1SD increase	3.85 (0.46), 96	−0.08	[-0.17, 0.01]	0.093
Number of IOP-lowering medications before DMEK [counts]	[1] 0	3.88 (0.45), 84	Reference	–	0.165
	[2] 1	3.79 (0.56), 4	−0.09	[-0.56, 0.37]	
	[3] 2	3.59 (0.56), 6	−0.30	[-0.68, 0.09]	
	[4] 3	3.31 (0.13), 2	−0.57	[-1.22, 0.08]	
Axial length [mm]	1SD increase	3.85 (0.46), 96	0.03	[-0.06, 0.13]	0.472
Pre-DMEK VA [logMAR]	1SD increase	3.85 (0.46), 96	−0.07	[-0.17, 0.02]	0.133
VA at last visit [logMAR]	1SD increase	3.85 (0.46), 96	−0.05	[-0.15, 0.04]	0.277
Preoperative donor ECD [cells/mm²]	1SD increase	3.85 (0.46), 96	0.04	[-0.06, 0.13]	0.416
ECD at 12 months [cells/mm²]	1SD increase	3.85 (0.46), 96	−0.01	[-0.10, 0.09]	0.893
Loss of ECD at 12 months [%]	1SD increase	3.85 (0.46), 96	0.01	[-0.08, 0.11]	0.801
ECD at last visit [cells/mm²]	1SD increase	3.87 (0.45), 92	0.04	[-0.06, 0.13]	0.434
Pre-DMEK CCT [µm]	1SD increase	3.85 (0.46), 96	−0.08	[-0.17, 0.02]	0.109
CCT at last visit [µm]	1SD increase	3.85 (0.46), 96	−0.10	[-0.20, -0.01]	0.027
Dark spots on specular microscopy	[1] No	3.91 (0.50), 56	Reference	–	0.149
	[2] Yes	3.77 (0.41), 40	−0.14	[-0.33, 0.05]	
CME onset	[1] No	3.88 (0.46), 83	Reference	–	0.143
	[2] Yes	3.67 (0.45), 13	−0.20	[-0.48, 0.07]	
Graft failure	[1] No	3.87 (0.46), 87	Reference	–	0.142
	[2] Yes	3.63 (0.46), 9	−0.24	[-0.56, 0.08]	

Standard deviations (SD): Age = 8.2 years, Pre-DMEK IOP = 3.7 mmHg, Axial length = 1.67 mm, Pre-DMEK VA = 0.485 logMAR, VA at last visit = 0.345 logMAR, Preoperative donor ECD = 205.0 cells/mm², ECD at 12 months = 479.8 cells/mm², Loss of ECD at 12 months = 16.88%, ECD at last visit = 413.4 cells/mm², Pre-DMEK CCT = 96.3 µm, CCT at last visit = 53.9 µm.

Abbreviations: ACD = anterior chamber depth; ALI = argon laser iridotomy; VA = visual acuity; BK = bullous keratopathy; CCT = central corneal thickness; CI = confidence interval; CME = cystoid macular edema; ECD = endothelial cell density; FECD = Fuchs endothelial corneal dystrophy; IOP = intraocular pressure; logMAR = logarithm of the minimum angle of resolution; PACD = primary angle closure disease; shallow-AC = shallow anterior chamber; PEX = pseudoexfoliation; PKP = penetrating keratoplasty; SD = standard deviation.

### Comparison between the shallow anterior chamber phenotype group and FECD

S4 Table in S1 File compares the shallow anterior chamber phenotype and FECD groups. Eyes with the shallow anterior chamber phenotype group had significantly lower pre- (1.93 ± 0.40 mm vs. 2.28 ± 0.27 mm, P < 0.001) and post-cataract surgery ACD (3.66 ± 0.33 mm vs. 4.08 ± 0.44 mm, P < 0.001), shorter axial length (22.50 ± 0.78 mm vs. 23.30 ± 1.18 mm, P = 0.006), and higher Pre-DMEK CCT (709.8 ± 97.0 µm vs. 659.3 ± 89.6 µm, P = 0.041) than eyes with FECD. Final BCVA was significantly better in the shallow anterior chamber phenotype group than in the FECD group (0.161 ± 0.426 logMAR vs. 0.185 ± 0.346 logMAR, P = 0.041).

### Comparison between PACD/ALI and shallow-AC subgroups

S5 Table in S1 File compares the PACD/ALI and shallow-AC subgroups. Eyes in the PACD/ALI subgroup had significantly lower pre-cataract surgery ACD (1.72 ± 0.24 mm vs. 2.20 ± 0.43 mm, P = 0.005) and higher Pre-DMEK CCT (741.3 ± 103.9 µm vs. 653.1 ± 47.4 µm, P = 0.014) than those in the shallow-AC subgroup; however, post-cataract surgery ACD did not differ significantly between the groups (3.61 ± 0.29 mm vs. 3.75 ± 0.38 mm, P = 0.265). No significant differences were observed in endothelial parameters or visual outcomes.

### Kaplan–Meier analyses

**Fig 3:**
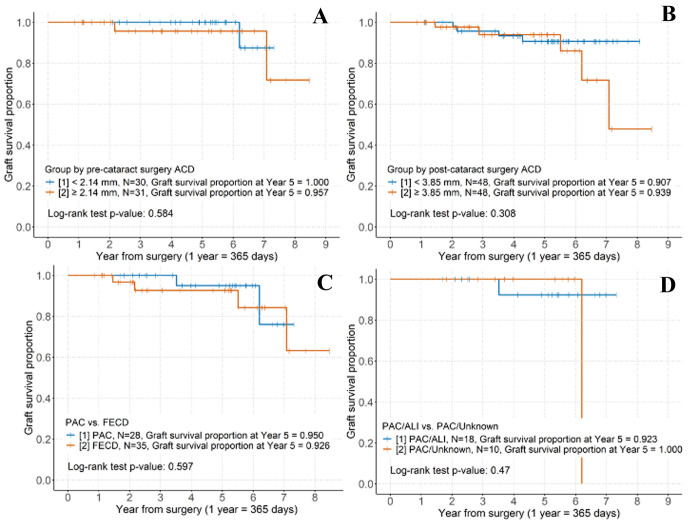
Kaplan–Meier graft survival curves stratified by subgroup. Kaplan–Meier graft survival curves stratified by (a; upper-left panel) pre-cataract ACD (low vs high), (b; upper-right panel) post-cataract ACD, (c; lower-left panel) etiology (the shallow anterior chamber phenotype vs. FECD), and (d; lower-right panel) subgroup (PACD/ALI vs. shallow-AC). Graft survival did not differ based on anterior chamber depth (ACD) measured before cataract surgery or after cataract surgery. Survival was also comparable between the shallow anterior chamber phenotype and FECD groups. Within the shallow anterior chamber phenotype group, graft survival was similar between the PACD/ALI and shallow-AC subgroups. **Abbreviations:** ACD = anterior chamber depth; PACD = primary angle closure disease; FECD = Fuchs endothelial corneal dystrophy; ALI = argon laser iridotomy.

In Kaplan–Meier analyses, graft survival did not differ based on ACD measured before cataract surgery (low < 2.14 mm vs. high ≥2.14 mm; log-rank P = 0.584; 5-year survival: 100% vs. 95.7%; Fig 3A) or after cataract surgery (low < 3.85 mm vs. high ≥3.85 mm; P = 0.308; 5-year survival: 90.7% vs. 93.9%; Fig 3B). Similarly, survival was comparable between the shallow anterior chamber phenotype and FECD groups (P = 0.597; 5-year survival: 95.0% vs. 92.6%; Fig 3C). Within the shallow anterior chamber phenotype group, graft survival was similar between the PACD/ALI and shallow-AC subgroups (P = 0.47; 5-year survival: 92.3% vs. 100%; Fig 3D), although the shallow-AC subgroup had a smaller sample and shorter follow-up (n = 10).

## Discussion

In this study, eyes in the shallow anterior chamber phenotype group undergoing DMEK had characteristically shallower ACD both before and after cataract surgery, shorter axial length, and greater pre-DMEK CCT than eyes with FECD. After cataract extraction, the relative ranking of ACD tended to persist. In addition, the association of shallower ACD with female sex and angle-closure–related disease, including APAC, is broadly consistent with prior reports [16,17]. Together, these findings suggest that eyes with the shallow anterior chamber phenotype may have anterior segment characteristics that differ from those of eyes with FECD. Despite these anatomic differences, graft survival after DMEK was favorable and did not differ across pre- or post-cataract surgery ACD strata, between the shallow anterior chamber phenotype and FECD groups, or between the PACD/ALI and shallow-AC subgroups. Graft survival in the shallow-AC subgroup appeared broadly similar to that in the PACD/ALI subgroup, although the sample size was smaller and follow-up was shorter.

We identified the shallow-AC subgroup that developed endothelial compromise in the anatomical context of a shallow anterior chamber alone, without any history of APAC or ALI, and compared this subgroup with the PACD/ALI subgroup. The shallow-AC subgroup may represent a clinical phenotype in which endothelial failure can occur in the absence of overt injurious events, thereby supporting the possibility that a shallow anterior chamber contributes to endothelial vulnerability. Roy-Chowdhury et al. demonstrated the feasibility of DMEK in eyes with primary angle closure suspects in Germany [9]. Our multicenter Japanese study builds on these observations by suggesting that a shallow anterior chamber may itself represent an anatomical risk phenotype for endothelial failure even without a history of APAC or ALI. Recently, Kusumi et al. reported prominent corneal endothelial cell density loss in eyes with primary angle-closure disease, including primary angle-closure suspect eyes without a history of acute angle-closure attacks or laser iridotomy. They further showed that a larger corneal curvature radius, reflecting a flatter corneal configuration, was associated with reduced corneal endothelial cell density, whereas ACD and angle parameters were not significantly different between eyes with decreased and normal endothelial cell density [18]. Although our shallow-AC subgroup was not defined as PACD/PACS based on uniform gonioscopic criteria, their findings support the broader concept that anterior segment morphology may contribute to endothelial vulnerability even in the absence of acute attacks or prior laser iridotomy.

Compared with the FECD group, eyes in the shallow anterior chamber phenotype group had significantly shallower ACD both before and after cataract surgery, shorter axial length, and greater pre-DMEK CCT. Specular microscopy (Fig 2A and 2B) did not show the guttate pattern typical of FECD. Additionally, in vivo confocal microscopy (Fig 2F) demonstrated endothelial dropout with fibrous or reflective deposits in the shallow-AC subgroup, rather than findings suggestive of the guttate-dominant pattern typically seen in FECD [15]. A transmission electron micrograph of the Descemet membrane from a shallow-AC case (Fig 2G) showed a relatively smooth posterior surface without the nodular guttae typically seen in FECD [19]. Within the shallow anterior chamber phenotype group, the shallow-AC subgroup had significantly deeper pre-cataract surgery ACD and a lower pre-DMEK CCT than the PACD/ALI subgroup, indicating less marked anterior chamber shallowing. These findings also raise the possibility that posterior stromal morphology may provide additional clues to endothelial dysfunction in this setting. Recent studies have suggested that posterior stromal ripples (PSR) are not merely descriptive findings on anterior segment optical coherence tomography (AS-OCT), but structural biomarkers of stromal overhydration and lamellar micro-distortion after DMEK [20]. In particular, preoperative PSR have been associated with slower visual recovery, whereas postoperative PSR have been strongly associated with rebubbling and graft instability [20,21]. Although PSR were not systematically assessed in the present study, the posterior surface irregularity shown in Fig 2C may be compatible with this concept.

Regarding post-DMEK graft survival, no significant differences were observed between eyes stratified by ACD measured before or after cataract surgery, between the shallow anterior chamber phenotype and FECD groups, or between shallow-AC and PACD/ALI. All groups achieved a favorable 5-year survival. Prior reports have noted that, in eyes with shallow chambers, intraoperative vitreous pressure tends to rise, the anterior chamber narrows further, and graft insertion is impeded; under these conditions, the graft can readily buckle or fold and may even be expelled through the wound [12]. In the present cohort, surgeons individualized perioperative management of shallow chambers, prioritizing pressure control and controlled graft unfolding. When an anterior chamber maintainer, a low-viscosity OVD, or both were used for insertion, they were carefully removed to avoid compromising graft adhesion. With such tailored perioperative management, favorable long-term DMEK outcomes may be achievable in shallow-chamber eyes, particularly when pressure management and graft manipulation are adapted to the anterior segment configuration.

Recent AS-OCT-based studies have suggested that subclinical postoperative inflammation may influence graft behavior after DMEK. In particular, higher postoperative inflammatory load, reflected by the aqueous-to-air relative intensity (ARI) index and hyperreflective dots, has been associated with PSR, and postoperative PSR have been strongly associated with rebubbling and graft instability [20,21]. Although neither PSR nor postoperative inflammatory activity was systematically quantified in the present study, these findings raise the possibility that subclinical inflammation may contribute to variability in postoperative behavior despite apparently uneventful surgery.

In addition to postoperative inflammatory factors, previous studies using models of shallow anterior chambers after ALI have shown increased aqueous flow velocity through the iridotomy window, and shear stress from the altered flow can reduce endothelial cell counts. These findings support the concept that an aqueous jet from the laser iridotomy window may injure the corneal endothelium [22,23]. To our knowledge, aqueous dynamics in eyes with shallow chambers but without prior ALI have not been modeled; however, even in the absence of an iridotomy, preferential flow patterns and locally increased velocity may plausibly impose shear stress on the endothelium.

A mean diurnal IOP fluctuation of approximately 5.99 mmHg has been reported in PAC/PACS eyes, and dark-room provocative testing can elicit IOP elevations of ≥8 mmHg in some cases. Such eyes tend to have shallower anterior chambers and more angle-closure quadrants [24,25]. Although these observations were derived from PAC/PACS eyes, intermittent IOP elevation and episodic iridocorneal contact may still represent biologically plausible mechanisms of endothelial injury in eyes with a shallow anterior chamber configuration.

The shallow-AC subgroup may be viewed as a secondary endothelial-injury phenotype associated with anatomic narrowing of the anterior chamber. Clinically, the absence of an acute attack or ALI history should not be equated with safety; rather, a shallow anterior chamber may still represent an anatomical marker of risk for endothelial damage. Regular monitoring of ECD and CCT in eyes with shallow chambers may facilitate early detection of endothelial compromise.

From a mechanistic perspective, endothelial vulnerability in eyes with a shallow anterior chamber may be better understood not as the consequence of a single acute event, but as the cumulative effect of chronic microstress related to anterior segment crowding. In such eyes, a compact anterior segment configuration may predispose to localized alterations in aqueous flow and micro-shear forces, while intermittent intraocular pressure elevation, episodic iridocorneal contact, and low-grade inflammation may further contribute to chronic endothelial stress. This framework may help explain the biological plausibility of endothelial decompensation even in the shallow-AC subgroup without a documented history of APAC or ALI.

### Limitations

The shallow-AC subgroup was small (n = 10) and had relatively short follow-up, highlighting the need for further case accumulation; this may have limited the power to detect modest between-group differences in some analyses, including graft survival. Most analyses were univariable and did not fully control for confounders such as age, sex, axial length, CCT, and perioperative factors, and selection bias inherent to the retrospective design could not be excluded. Therefore, in this context, a shallow anterior chamber should be interpreted as a potential marker of the angle-closure spectrum, while confirmation of an independent effect will require larger studies. Thus, the present study does not establish a definite causal link between shallow anterior chamber and endothelial cell loss. These findings should be regarded as exploratory and hypothesis-generating until validated in larger cohorts with longer follow-up and multivariable adjustment.

## Conclusion

Our findings suggest that a shallow anterior chamber configuration without any documented history of APAC or ALI may be associated with corneal endothelial decompensation. Long-term outcomes after DMEK appeared favorable in this cohort, and appropriate preoperative evaluation and perioperative optimization may help achieve good postoperative outcomes in eyes with a shallow anterior chamber phenotype, including those with and without prior ALI or APAC. Future studies with larger sample sizes should develop operational definitions to enable early identification of this phenotype and to optimize intervention strategies.

## Supporting information

S1 File Supplementary Files S1-S4. **Contains: S1 Dataset.** The dataset includes all information used for this study. **S2 Table.** Cataract surgery-related characteristics in eyes with the shallow anterior chamber phenotype. **S3 Fig. Scatter plot with density contours showing pre- versus post-cataract surgery anterior chamber depth (ACD) values**. A significant positive correlation was observed (Pearson’s r = 0.474, P < 0.001), indicating that eyes with lower pre-cataract surgery ACD tended to have relatively lower post-cataract surgery ACD. **S4 Table.** Pre- and post-operative characteristics and measurements for the shallow anterior chamber phenotype and FECD groups. **S5 Table.** Pre- and post-operative characteristics and measurements for PACD/ALI and shallow-AC groups.
(PDF)
