# Supplementary material for: Bullous keratopathy associated with a shallow anterior chamber: An anatomical risk phenotype for corneal endothelial decompensation
Source: PLoS One. 2026 Jul 7;21(7):e0353272. doi: 10.1371/journal.pone.0353272 (PMC13340822; doi:10.1371/journal.pone.0353272)
Supplement: S1 File — Contains: S1 Dataset. The dataset includes all information used for this study. S2 Table. Cataract surgery-related characteristics in eyes with the shallow anterior chamber phenotype. S3 Fig. Scatter plot with density contours showing pre- versus post-cataract surgery anterior chamber depth (ACD) values. A significant positive correlation was observed (Pearson’s r = 0.474, P < 0.001), indicating that eyes with lower pre-cataract surgery ACD tended to have relatively lower post-cataract surgery ACD. S4 Table. Pre- and post-operative characteristics and measurements for the shallow anterior chamber phenotype and FECD groups. S5 Table. Pre- and post-operative characteristics and measurements for PACD/ALI and shallow-AC groups. (PDF) [file pone.0353272.s001.pdf]

S1 Dataset

Abbreviations and notes

Abbreviations and notes: ABK, aphakic bullous keratopathy; ACD, anterior chamber depth; ALI, argon laser iridotomy; APAC, acute primary angle closure; BK, bullous keratopathy; CCT, central corneal thickness; CME, cystoid macular edema; DMEK, Descemet membrane endothelial keratoplasty; DM, Descemet membrane; ECD, endothelial cell density; FECD, Fuchs endothelial corneal dystrophy; IOL, intraocular lens; IOP, intraocular pressure; LI, laser iridotomy; logMAR, logarithm of the minimum angle of resolution; NA, not applicable; NE, not evaluable; NR, not recorded; PACD, primary angle-closure disease; PBK, pseudophakic bullous keratopathy; PEX, pseudoexfoliation; PKP, penetrating keratoplasty; STTA, sub-Tenon's triamcinolone acetonide injection; VA, visual acuity.

For categorical variables, "Yes" and "No" indicate the presence or absence of the corresponding finding or procedure. "NA" indicates that the variable was not applicable, "NE" indicates that the variable could not be reliably evaluated, and "NR" indicates that the information was not recorded in the available medical records.

Abbreviations: ALI, argon laser iridotomy; BK, bullous keratopathy; CDE, cumulative dissipated energy; DMEK, Descemet membrane endothelial keratoplasty; ECD, endothelial cell density; IOL, intraocular lens; NA, not applicable; NE, not evaluable; NR, not recorded; PACD, primary angle-closure disease; shallow-AC, shallow anterior chamber.

Negative values for the interval from cataract surgery to corneal edema confirmation indicate that corneal edema or endothelial decompensation had already been documented before cataract surgery.

1	R	BK	PACD(ALI)	F	62	0	1	0.9	685	1	580	647	0.1	1	1.2	1.2	1	1	757	526	546	553	552	554	2804	1370	1049	530	512	537	N	62.58916	no	No	69
2	L	BK	PEX	F	74	0	0			0.7	576	733	0.1	0.9	0.7	0.6p	0.4p	0.4p	757	557	576	601	589	639	2517	929	733	446	534	403	N	70.87803	no	No	75
3	R	FECD	FECD	F	74	1(infection)	0	0.1	722	0.01	687	NA	0.3	0.5	0.5	0.1	0.01	0.01	550	519	522	576	659	687	2050	979	679	723	NA	NA	N	66.87805	no	No	68
4	R	FECD	FECD	F	76	0	0			0.5	580	797	0.4	0.7	0.7	0.7	0.7	0.7	585	519	557	553	551	595	2682	1261	1092	975	887	731	Y	77.92692	no	No	53
5	R	BK	PBK	F	70	1	1	0.6	607	1.2	518	631	0.4	1	0.8	0.2	0.9(reope)	1.2(reope)	670	523	523	700	509(reope)	518(reope)	2530	929	465	666	1058(reope)	635(reope)	N	81.62055	no	No	70
6	L	BK	ABK	F	56	0	0			0.8	563	722	0.3	1	1	1.0p	1.0p	NA	757	553	569	578	591	NA	2582	1345	846	746	727	NA	Y	67.2347	no	Yes	70
7	R	BK	PACD(ALI)	M	73	0	0			1	502	770	0.05	0.9	1	1	NA	NA	937	469	502	527	NA	NA	2020	1045	770	677	NA	NA	Y	61.88119	no	No	53
8	R	uveitis	uveitis	M	57	0	0			0.6	501	1055	0.2	1	1	1	NA	NA	718	483	489	498	NA	519	2450	1370	1060	805	NA	1016	Y	56.73469	no	Yes	68
9	L	FECD	FECD	F	60	0	0			1	539	985	0.6	1	1.2	1.2	1.2	1.2p	558	549	547	543	542	541	2634	1410	1159	1255	1718	1030	Y	55.99848	no	No	68
10	L	FECD	FECD	F	83	0	0			0.9	474	479	0.02	0.8	0.9	0.9	NA	NA	723	455	481	484	NA	NA	2506	1515	893	479	NA	NA	N	64.36552	no	No	68
11	L	BK	ABK	F	69	1	0	0.02	727	0.1	637	NA	0.06	0.7	0.4	0.1	0.1	0.1	734	590	606	639	643	637	2722	1025	515	NA	NA	NA	Y	81.08009	no	No	66
12	L	FECD	FECD	F	76	1	1	0.04	760	0.4	630	NA	0.2	0.6	0.7	0.5	0.4	0.4	922	493	533	534	564	630	3083	1686	752	699	450	NA	Y	75.60817	no	No	70
13	R	BK	PACD(ALI)	F	71	0	0			0.9	578	707	0.02	0.9	0.9	0.9	NA	NA	911	531	546	578	NA	NA	2336	950	736	707	NA	NA	N	68.49315	no	No	68
14	R	FECD	FECD	F	83	0	0			0.7	515	443	0.01	0.8	0.8	0.7	NA	NA	795	437	488	515	NA	NA	2320	945	710	443	NA	NA	N	69.39655	no	No	68
15	L	BK	PACD(ALI)	F	71	0	0			1.2	563	734	0.3	1.2	1.2	1.2	NA	NA	722	540	540	563	NA	NA	2857	1420	1000	734	NA	NA	N	64.99825	no	No	62
16	L	BK	PEX	M	79	0	0			0.4	606	1110	0.05	0.7	0.6	0.5p	0.4p	NA	840	526	543	617	606	NA	3227	2139	1618	887	1110	NA	N	49.86055	no	Yes	75
17	R	BK	PEX	F	75	0	0			0.8	541	847	0.3	0.8	0.8	0.8p	1	NA	757	514	541	551	575	NA	3118	1386	847	692	667	NA	N	72.83515	no	No	75
18	L	BK	PBK	M	69	0	0			1.2	473	675	1.2	1.2	1.2	1.2	NA	1.2	581	456	461	501	NA	527	3313	2213	1836	1199	NA	663	N	44.58195	no	No	69
19	R	FECD	FECD	F	73	0	0			0.8	461	1934	0.05	0.8	0.8	0.8p	0.6	0.8p	744	443	445	460	458	457	2513	2324	2169	1326	838	627	N	13.68882	no	Yes	69
20	L	BK	PACD(ALI)	F	78	0	0			0.9	532	667	0.2	1	1	1	0.8	0.9	571	475	473	473	510	532	2607	1328	787	659	552	641	N	69.81204	no	No	61
21	R	BK	PACD(ALI)	F	81	0	0			0.7	574	940	0.02	1	1	1.0p	0.9p	1	760	467	470	474	470	517	2530	1297	1043	857	598	791	N	58.7747	no	No	72
22	R	BK	PACD(ALI)	F	77	0	0			0.8	486	1672	0.3	1	1	0.7	NA	NA	621	532	529	517	NA	NA	2694	1905	1776	1610	NA	NA	N	34.07572	no	No	73
23	R	BK	shallow-AC	F	69	1	1	0.01	760	1.2	583	1094	0.3	1	1.2	1.2	1.2	1.2	745	575	576	578	590	583	2743	2364	2257	2128	1838	1094	Y	17.71783	no	No	65
24	L	BK	PBK	F	82	0	0			0.8	548	601	0.03	1	1	1.2	NA	NA	732	525	530	541	NA	557	2428	948	770	702	NA	NA	N	68.28666	no	No	62
26	L	BK	shallow-AC	F	69	0	0			0.8	585	787	0.4	1.2	1.2	1.2	1.2	1	624	585	582	594	603	NA	2743	1324	1255	997	672	838	Y	54.24717	no	Yes	60
27	R	BK	PBK	F	82	0	0			1	574	877	0.05	1	1	1	1	1	667	516	518	529	NA	562	2584	1613	1043	857	NA	NA	Y	59.63622	no	No	70
28	L	FECD	FECD	F	73	0	0			0.7	502	1785	0.3	0.8	0.7	0.7	0.7p	0.6p	690	493	486	504	502	495	2783	2216	1988	2053	1873	1852	N	28.5663	no	No	73
29	L	BK	ABK	F	74	0	0			0.4	528	1026	0.1	0.5	0.5	0. 4 p	NA	NA	822	528	526	528	NA	NA	2675	2119	1176	1026	NA	NA	Y	56.03738	no	No	75
30	L	FECD	FECD	M	64	0	0			1.2	468	1127	0.1	0.8	1.2	1.2	1.2	NA	675	456	466	470	468	NA	2762	1658	1546	1229	1127	NA	N	44.02607	no	No	66
31	R	BK	ABK	M	79	0	0			0.3	506	887	0.04	0.4	0.4	0.3	0.3p	NA	956	483	485	504	506	NA	2634	1206	1087	923	887	NA	N	58.73197	no	No	69
32	R	FECD	FECD	M	74	1	0	0.8	640	0.9	607	609	0.05	1.2	1.2	1.2	1.2	1.2	757	508	531	538	535	559	2762	2002	1274	743	464	422	Y	53.874	no	Yes	66
33	L	BK	shallow-AC	F	75	0	0			0.1	507	911	0.4	0.7	0.7	0.1	0.1	NA	660	511	508	510	507	NA	2731	1730	1531	1220	911	NA	N	43.93995	no	No	65
34	R	FECD	FECD	F	79	0	0			0.9	530	1342	0.3	1.2	1.2	0.8	0.9	NA	632	526	543	525	530	NA	2767	1912	1818	1825	1342	NA	Y	34.29707	no	No	61
35	L	BK	ABK	F	79	0	0			1	543	1695	0.1	1.2	1.2	1.0p	NA	NA	626	544	556	543	NA	NA	2536	1892	2110	1695	NA	NA	N	16.79811	no	No	58
36	R	BK	shallow-AC	M	74	0	0			0.8	514	1235	0.1	0.8	0.8	0.7	0.4	NA	586	537	538	512	513	NA	2538	1493	1235	947	895	NA	N	51.33964	no	No	72
37	R	FECD	FECD	F	81	0	0			1.2	531	616	0.9	1.2	1.2	1.2	NA	NA	570	512	521	497	NA	NA	2646	2326	1847	1536	NA	NA	N	30.19652	no	No	67
38	L	FECD	FECD	M	74	0	0			0.5	441	1730	0.3	0.6	0.6	0.6	0.6	0.6	501	441	438	468	497	497	2967	2604	2519	1938	1757	1981	N	15.09943	no	No	65
39	L	BK	PEX	F	85	0	0			0.5	484	360	0.1	0.5	0.6	0.8	0.7p	0.8p	585	463	454	453	484	477	2582	485	464	457	508	872	Y	82.02943	no	No	65
40	R	BK	shallow-AC	F	75	0	0			0.9	511	1799	0.8	1.2	1	1.2	NA	NA	645	520	510	515	NA	NA	2632	1416	1502	1629	NA	NA	N	42.93313	no	No	70
41	L	Failed PKP	Failed PKP	M	54	0	1	0.7	660	1	534	871	0.4	1.2	1.2	1	1.2	1.2	655	491	499	505	515	523	3003	1093	1021	654	608	561	Y	66.00067	no	No	72
42	R	FECD	FECD	F	72	0	0			0.9	569	678	0.2	1.2	1.2	1.2	0.9	NA	713	529	530	540	555	NA	2506	1029	899	761	686	NA	Y	64.1261	no	No	66
43	R	BK	PEX	F	81	0	0			0.4	544	1246	0.02	0.8	1	0.8	0.8	0.8	749	539	544	599	560	549	2801	1629	1678	1387	1292	1246	N	40.09282	no	No	73
44	R	BK	PACD(ALI)	F	75	0	0			1.0	561	792	0.8	1	1.2	0.9	0.4p	NA	719	556	536	560	565	NA	2625	1457	1779	806	594	NA	N	32.22857	no	Yes	68
45	R	FECD	FECD	M	55	0	0			0.9	533	1044	0.8	0.8	1	0.9p	NA	NA	636	508	528	547	NA	577	2812	1497	1293	NA	NA	600	Y	54.01849	no	No	73
46	R	BK	PACD(ALI)	F	83	0	0			0.9	487	714	0.4	1	1	0.9p	NA	NA	592	466	483	487	NA	NA	2500	1215	1220	714	NA	NA	Y	51.2	no	No	73
47	L	BK	PACD(ALI)	F	77	0	0			1	458	390	0.3	0.8	0.9	0.9	NA	NA	704	445	457	460	NA	NA	2618	546	363	360	NA	NA	Y	86.13445	no	No	71
48	R	BK	PACD(ALI)	M	77	0	0			0.7	530	595	0.1	0.5	0.6	0.7	NA	NA	737	524	524	526	NA	NA	2519	1873	1082	641	NA	NA	Y	57.04645	no	Yes	71
49	R	BK	PBK	F	72	0	0			0.9	522	631	0.06	0.9	1	1	NA	NA	715	497	505	518	NA	NA	2530	1366	915	635	NA	NA	N	63.83399	no	No	70
50	R	Failed PKP	Failed PKP	M	69	0	0			1.2	430	1342	0.7	1	1	1.2	1.2	NA	547	398	402	426	430	NA	2860	2282	2083	1493	1342	NA	N	27.16783	no	No	70
51	R	FECD	FECD	M	64	0	0			1.2	481	1692	0.1	0.8	1.2	1. 2 p	NA	NA	675	456	466	481	NA	NA	2762	1658	1546	1692	NA	NA	N	44.02607	no	No	61
52	R	BK	PEX	F	85	0	0			0.8	452	711	0.1	0.5	0.7	0.8p	0.9p	0.8p	585	463	471	459	457	462	2582	1302	1232	1085	758	920	Y	52.28505	no	No	65
53	R	BK	PEX	M	89	0	0			0.7	502	802	0.3	0.7	0.7	0.7	NA	NA	577	508	492	502	NA	NA	2506	1642	1247	802	NA	NA	N	50.23943	no	No	67
54	R	BK	PACD(ALI)	F	77	0	0			1	440	844	0.3	1	0.9	1	NA	NA	692	460	457	440	NA	NA	2506	1241	1277	844	NA	NA	Y	49.0423	y	No	72
55	L	BK	PEX	F	75	0	0			0.9	494	1567	0.5	1	1	0.9p	NA	NA	671	509	499	494	NA	NA	2801	2083	1942	1567	NA	NA	N	30.66762	y	Yes	68
56	R	BK	PEX	M	75	0	0			0.7	458	1056	0.2	0.8	0.9	0.6	NA	NA	661	474	465	444	NA	NA	2513	1196	1067	1130	NA	NA	N	57.54079	y	No	65
57	R	BK	PEX	F	78	0	0			0.8	560	723	0.7	1	1	0.8	0. 8 p	NA	878	499	509	549	560	NA	2545	859\	832	402	723	NA	N	67.30845	y	No	59
58	L	FECD	FECD	F	73	0	0			0.9	518	622	0.3	0.7	0.8	0.9p	NA	NA	706	476	478	518	NA	NA	2959	1071	993	622	NA	NA	Y	66.44137	y	No	55
59	L	BK	ABK	F	83	0	0			0.1	457	805	0.1	0.2	0.3	NA	NA	NA	633	459	454	NA	NA	NA	2632	1149	934	836	749	649	N	64.51368	y	No	57
61	R	uveitis	uveitis	M	80	0	0			0.3	546	653	0.3	0.6	0.6	0.8	0.15	NA	645	528	531	536	548	NA	2546	1001	859	676	653	NA	Y	66.2608	y	No	70
62	L	BK	PACD(ALI)	F	76	0	0			0.7	567	639	0.7	1	1	0.9	NA	1	856	561	558	566	NA	596	2710	1821	1635	839	NA	538	N	39.6679	y	No	68
63	R	BK	PBK	F	68	0	0			1.2	496	861	0.1	0.8	0.8	1.2	NA	NA	687	486	493	505	490	492	2532	836	792	828	820	861	Y	68.72038	y	Yes	63
64	L	BK	shallow-AC	F	65	0	0			0.9	412	720	0.1	0.9	0.9	1.2	NA	NA	690	397	413	453	NA	463	2531	1476	1345	836	NA	720	N	46.85895	y	No	66
66	L	BK	PACD(ALI)	F	78	0	0			1.0	527	1062	0.3	1.2	1.2	1	NA	NA	753	538	521	531	534	538	2901	2297	2043	1581	1463	1105	N	29.57601	y	No	62
67	L	BK	PEX	M	71	1	1	0.01	661	0.7	572	750	0.2	0.9	0.9	0.9	0.7	NA	619	461	465	470	572	NA	2650	1645	1534	1032	750	NA	Y	42.11321	y	No	63
68	R	BK	PEX	M	84	0	0			0.8	522	834	0.1	0.7	0.8	0.15p	NA	NA	669	518	532	605	NA	NA	2625	1326	1126	806	NA	NA	Y	57.10476	y	No	70
69	R	FECD	FECD	M	84	0	0			0.8	496	1785	0.6	0.8	0.7	0.7p	0.8p	NA	601	481	482	489	489	NA	2704	2053	1953	1889	1785	NA	Y	27.77367	y	No	73
70	L	FECD	FECD	M	84	0	0			0.8	526	682	0.5	0.6	0.6	0.7	0.8	NA	611	521	521	532	526	NA	2532	970	667	946	632	NA	Y	73.65719	y	No	73
71	L	BK	shallow-AC	F	84	0	0			0.8	576	722	0.06	0.5	0.5	1.0p	0.8	NA	681	516	514	546	567	NA	2506	1205	1053	660	722	NA	Y	57.98085	y	No	61
72	R	BK	PEX	M	71	0	0			0.5	497	465	0.2	0.9	0.9	0.9	NA	NA	572	483	499	541	NA	NA	2650	772	667	792	NA	NA	Y	74.83019	y	Yes	63
74	L	BK	ABK	F	75	0	0			0.4	511	933	0.3	0.4	0.4	0.4	NA	NA	613	507	524	500	NA	NA	2725	1497	1064	850	NA	NA	Y	60.95413	y	No	66
75	L	BK	PACD(ALI)	F	83	0	0			0.9	543	1235	0.04	0.9	0.9	NA	NA	NA	857	558	549	NA	NA	NA	2506	1235	1020	NA	NA	NA	N	59.29769	y	No	70
76	L	BK	PACD(ALI)	F	74	1	1	0.01	769	20cm/n.d.	767	NA	0.01	1	1	NA	20cm/n.d.	NA	822	481	491	NA	767	NA	2695	1185	947	NA	NA	NA	Y	64.86085	y	Yes	65
77	R	BK	shallow-AC	F	79	0	0			1.2	478	1548	0.7	1	1	1.2	NA	NA	678	483	481	483	NA	NA	2632	1621	1429	1548	NA	NA	Y	45.70669	y	No	75
78	R	FECD	FECD	M	63	0	0			0.7	513	1138	0.15	0.7	0.8	0.7p	NA	NA	690	501	498	513	NA	NA	2519	1209	1136	1138	NA	NA	N	54.90274	y	No	69
79	R	birth injury	birth injury	M	66	0	0			1	507	2532	0.3	1	1	NA	NA	NA	606	504	507	NA	NA	NA	3003	2500	2432	NA	NA	NA	N	19.01432	y	No	54
80	R	BK	shallow-AC	F	84	0	0			1	469	964	0.3	0.9	1	NA	NA	NA	609	538	521	NA	NA	NA	2681	1201	1042	NA	NA	NA	N	61.13391	y	No	64
81	R	BK	PACD(ALI)	F	70	0	0			1	481	1104	0.3	0.7	0.9	1	1	NA	690	464	474	NA	NA	NA	2645	952	827	NA	804	NA	Y	68.73346	y	No	63
82	L	FECD	FECD	F	74	1	1	0.04	613	0.6	574	804	0.5	0.6	0.6	NA	NA	NA	604	584	577	574	NA	NA	2524	908	804	NA	NA	NA	Y	68.1458	y	Yes	47
83	R	FECD	FECD	F	84	0	0			0.8	514	631	0.2	0.6	0.7	0.7	0.7	NA	740	506	513	521	528	NA	2534	765	649	707	631	NA	N	74.38832	y	No	70
84	R	FECD	FECD	f	61	0	0			1	522	691	0.4	1	1.2	NA	NA	NA	652	506	519	NA	529	NA	2865	982	882	NA	703	NA	Y	69.21466	y	No	72
85	L	FECD	FECD	F	61	0	0			0.9	581	622	0.4	0.9	0.9	NA	NA	NA	626	565	557	NA	562	NA	2619	839	578	NA	622	NA	N	77.93051	y	No	55
86	L	FECD	FECD	F	55	0	0			1	501	780	1	1	1.2	NA	NA	NA	566	503	503	NA	NA	NA	2558	1158	876	856	NA	NA	N	65.7545	y	No	47
87	L	FECD	FECD	F	84	0	0			0.7	497	588	0.3	0.5	0.6	NA	NA	NA	561	488	492	NA	NA	NA	2821	802	647	NA	NA	NA	N	77.06487	y	No	62
88	R	FECD	FECD	F	87	0	0			0.4	512	726	0.2	0.5	0.4	NA	NA	NA	553	523	508	NA	NA	NA	2404	777	726	NA	NA	NA	Y	69.80033	y	No	64
89	R	FECD	FECD	F	84	0	0			0.3	565	660	0.2	0.4	0.6	NA	NA	NA	721	515	567	NA	NA	NA	2907	1003	998	NA	NA	NA	N	65.66907	y	No	64
90	L	FECD	FECD	F	87	0	0			0.5	515	1336	0.1	0.6	0.5	NA	NA	NA	556	556	508	NA	NA	NA	2941	1336	1336	NA	NA	NA	N	54.57327	y	No	67
91	R	FECD	FECD	F	73	0	0			0.7	481	829	0.3	1.2	1	NA	NA	NA	729	467	474	NA	NA	NA	2581	1128	829	NA	NA	NA	N	67.88067	y	No	71
92	L	BK	shallow-AC	F	74	0	0			1.2	539	853	1	1.2	1.2	1.2	NA	NA	613	541	538	593	NA	NA	2564	918	893	856	NA	NA	N	65.17161	y	No	51
93	L	BK	PACD(ALI)	F	86	0	0			0.2	499	937	0.8	0.4	0.5	NA	NA	NA	643	492	492	NA	NA	NA	2415	886	876	NA	NA	NA	Y	63.72671	y	No	57
94	R	FECD	FECD	F	81	0	0			0.6	483	885	0.01	0.1	0.8	NA	NA	NA	812	480	480	NA	NA	NA	2962	1579	885	NA	NA	NA	N	70.12154	y	No	62
95	R	birth injury	birth injury	F	58	0	0			0.8	531	1501	0.1	0.1	0.8	NA	NA	NA	868	525	525	NA	NA	NA	2681	1984	1583	NA	NA	NA	N	40.95487	y	No	60
96	R	FECD	FECD	M	71	0	0			1	484	1898	0.6	0.6	0.6	NA	NA	NA	587	481	481	NA	NA	NA	2809	1898	1722	NA	NA	NA	N	38.69705	y	No	73
97	L	BK	PBK	F	85	0	0			0.7	481	1292	0.05	0.15	0.8	NA	NA	NA	656	479	479	NA	NA	NA	2577	1552	1516	NA	NA	NA	N	41.17191	y	No	64
98	L	FECD	FECD	F	85	0	0			0.7	515	823	0.07	0.5	0.5	NA	NA	NA	670	516	516	NA	NA	NA	2500	1059	589	NA	NA	NA	Y	76.44	y	No	38
99	R	FECD	FECD	M	79	0	0			0.5	592	1564	0.2	0.4	0.5	NA	NA	NA	694	593	595	NA	NA	NA	2663	1564	1133	NA	NA	NA	N	57.454	y	No	56

Number	Eye	Etiology	Etiology-2	Interval_from_cataract_surgery_to_corneal_edema_confirmation_days	CDE	IOL_type	IOL_position
1	R	BK	PACD(ALI)	57	NR	YA-60BBR	in the bag
7	R	BK	PACD(ALI)	-22	NR	NX-60	in the bag
13	R	BK	PACD(ALI)	-76	NR	NX-60	in the bag
15	L	BK	PACD(ALI)	12	NR	NR	in the bag
20	L	BK	PACD(ALI)	-30	NR	ZCB00V	in the bag
21	R	BK	PACD(ALI)	-47	NR	NX-60	in the bag
22	R	BK	PACD(ALI)	-30	NR	NX-60	in the bag
23	R	BK	shallow-AC	-47	NR	KS-SP	in the bag
26	L	BK	shallow-AC	-30	NR	AN6KA	in the bag
33	L	BK	shallow-AC	-30	NR	KS-SP	in the bag
36	R	BK	shallow-AC	-27	NR	NX-60	in the bag
40	R	BK	shallow-AC	-27	NR	NX-60	in the bag
44	R	BK	PACD(ALI)	-30	NR	NX-60	in the bag
46	R	BK	PACD(ALI)	-58	NR	NX-60	in the bag
47	L	BK	PACD(ALI)	-41	NR	NX-60	in the bag
48	R	BK	PACD(ALI)	1	NR	NR	in the bag
54	R	BK	PACD(ALI)	-202	NR	NX-60	in the bag
62	L	BK	PACD(ALI)	-60	NR	NX-60	in the bag
64	L	BK	shallow-AC	-121	NR	KS-SP	in the bag
66	L	BK	PACD(ALI)	-75	NR	NX-60	in the bag
71	L	BK	shallow-AC	-62	NR	AN6KA	in the bag
75	L	BK	PACD(ALI)	-55	NR	NX-60	in the bag
76	L	BK	PACD(ALI)	NR	NR	NR	in the bag
77	R	BK	shallow-AC	-60	NR	NX-60	in the bag
80	R	BK	shallow-AC	-20	NR	AN6KA	in the bag
81	R	BK	PACD(ALI)	-20	NR	AN6KA	in the bag
92	L	BK	shallow-AC	NR	NR	NR	NR
93	L	BK	PACD(ALI)	NR	NR	AU6KA	in the bag

Interval_from_ALI_to_cataract_surgery_days	Pre_cataract_surgery_ECD	Clinical_findings_suggestive_of_viral_endotheliitis	Aqueous_humor_test_result	Planned_staged_cataract_surgery_before_DMEK
NR	NR	no	NA	No
NR	562	no	NA	Yes
NR	NE	no	NA	Yes
NR	583	no	NA	No
6696	NE	no	NA	Yes
2923	NE	no	NA	Yes
NR	569	no	NA	Yes
NA	356	no	NA	Yes
NA	NE	no	NA	Yes
NA	589	no	NA	Yes
NA	401	no	NA	Yes
NA	NE	no	NA	Yes
4555	NE	no	NA	Yes
6149	349	no	NA	Yes
NR	249	no	NA	Yes
NR	NR	no	NA	No
NR	344	no	NA	Yes
4902	NE	no	NA	Yes
NA	NE	no	NA	Yes
NR	386	no	NA	Yes
NA	NE	iritis	Negative	Yes
NR	NE	no	NA	Yes
NR	NE	no	NA	No
NA	NE	no	NA	Yes
NA	499	no	NA	Yes
3527	NE	no	NA	Yes
NA	NR	NR	NA	No
3017	514	no	NA	No

S2 Table. Cataract surgery-related characteristics in eyes with the shallow anterior chamber phenotype.

Variable	Overall eyes (N=28)
Planned staged cataract surgery before DMEK	22 (78.6%)
Non-planned or prior cataract surgery	6 (21.4%)
Interval from cataract surgery to corneal edema confirmation, days	-30 [-202, 57]
CDE available	0 (0.0%)
IOL type available	23 (82.1%)
IOL position available	27 (96.4%)
In-the-bag IOL fixation among evaluable eyes	27/27 (100%)
Sulcus IOL or anterior chamber IOL documented	0/27 (0.0%)
Interval from ALI to cataract surgery available	9 (32.1%)
Interval from ALI to cataract surgery, days	4555 [2923, 6696]
Recipient ECD before cataract surgery available	13 (46.4%)
Recipient ECD before cataract surgery, cells/mm²	499 [249, 589]
Recipient ECD before cataract surgery not evaluable	12 (42.9%)
Clinical findings suggestive of viral endotheliitis	1 (3.6%)
Aqueous humor testing performed	1 (3.6%)
Positive aqueous humor test result	0/1 (0.0%)

Values are presented as n (%), n/N (%), or median [range]. The interval from cataract surgery to corneal edema confirmation was calculated as the date of corneal edema confirmation minus the date of cataract surgery. Negative values indicate that corneal edema or endothelial decompensation had already been documented before cataract surgery. Planned staged cataract surgery before DMEK indicates eyes in which cataract surgery was performed after corneal endothelial decompensation or bullous keratopathy had already been recognized, as part of a planned staged surgical approach before DMEK.

Abbreviations: ALI, argon laser iridotomy; BK, bullous keratopathy; CDE, cumulative dissipated energy; DMEK, Descemet membrane endothelial keratoplasty; ECD, endothelial cell density; FECD, Fuchs endothelial corneal dystrophy; IOL, intraocular lens.

S3 Figure. Scatter plot with density contours showing pre- versus post-cataract surgery anterior chamber depth (ACD) values.

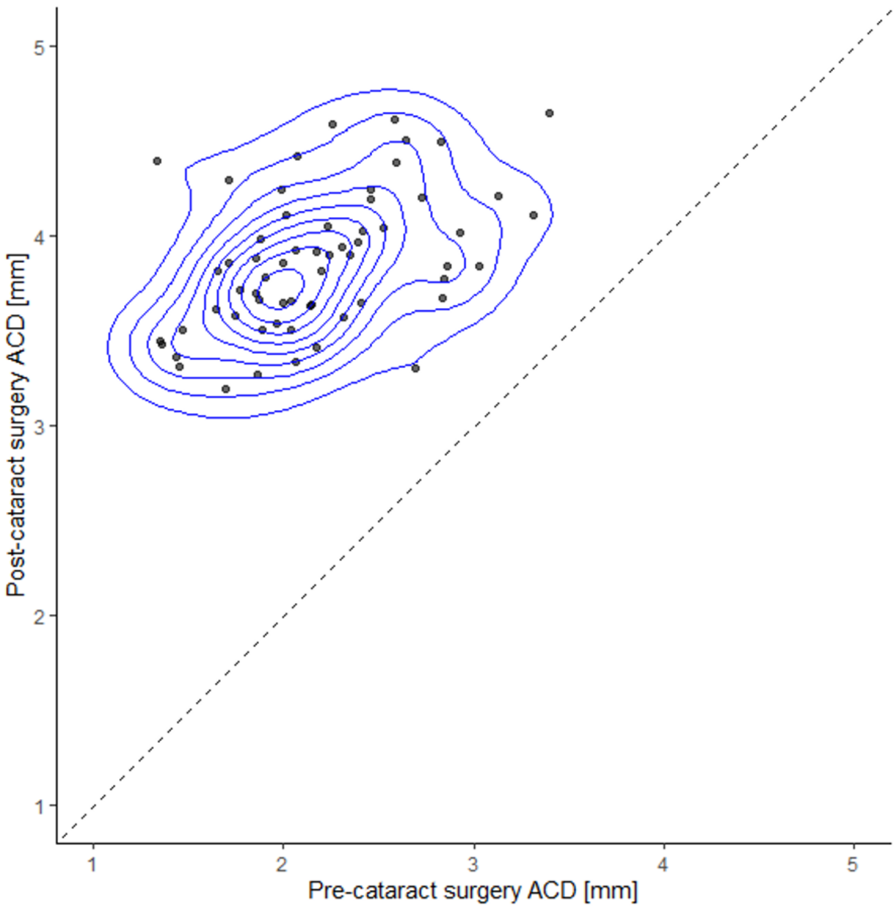

Scatter plot with density contours showing the relationship between pre- and post-cataract surgery anterior chamber depth (ACD) in eyes with available data (N = 61). The dashed diagonal line indicates equality between pre- and post-cataract surgery ACD values. Pre- and post-cataract surgery ACD values showed a moderate positive correlation (Pearson's r = 0.474, P < 0.001). Eyes with lower pre-cataract surgery ACD tended to have relatively lower post-cataract surgery ACD after cataract surgery.

Abbreviation: ACD, anterior chamber depth.

S4 Table. Pre- and post-operative characteristics and measurements for the shallow anterior chamber phenotype and FECD groups.

Item [unit]	Statistics Category	Group		Comparison between groups
		[1] The shallow anterior chamber phenotype (N=28)	[2] FECD (N=35)	
Age [years]	Mean (SD), N	75.6 (5.7), 28	74.6 (9.2), 35	Wilcoxon RS test
	Median [Range]	75.5 [62, 86]	74.0 [55, 87]	P=0.934
Sex	[1] Male	3 (10.7%)	11 (31.4%)	Fisher's exact test
	[2] Female	25 (89.3%)	24 (68.6%)	P=0.069
Pre-cataract surgery ACD [mm]	Mean (SD), N	1.93 (0.40), 28	2.28 (0.27), 35	Wilcoxon RS test
	Median [Range]	1.86 [1.4, 3.1]	2.25 [1.9, 2.9]	P<0.001
Post-cataract surgery ACD [mm]	Mean (SD), N	3.66 (0.33), 28	4.08 (0.44), 35	Wilcoxon RS test
	Median [Range]	3.63 [3.1, 4.3]	4.03 [3.3, 5.3]	P<0.001
Rebubbling	[1] No	22 (78.6%)	19 (54.3%)	Fisher's exact test
	[2] Yes	6 (21.4%)	16 (45.7%)	P=0.063
Pre-DMEK IOP [mmHg]	Mean (SD), N	12.7 (3.5), 28	11.3 (3.3), 35	Wilcoxon RS test
	Median [Range]	12.0 [7, 21]	11.0 [7, 21]	P=0.129
Number of IOP-lowering medications before DMEK [counts]	[1] 0	24 (85.7%)	32 (91.4%)	Wilcoxon RS test
	[2] 1	1 (3.6%)	1 (2.9%)	P=0.462
	[3] 2	2 (7.1%)	2 (5.7%)	
	[4] 3	1 (3.6%)	0 (0.0%)	
Axial length [mm]	Mean (SD), N	22.50 (0.78), 28	23.30 (1.18), 35	Wilcoxon RS test
	Median [Range]	22.54 [21.2, 24.5]	23.07 [21.8, 26.8]	P=0.006
Pre-DMEK VA [logMAR]	Mean (SD), N	0.714 (0.540), 28	0.698 (0.496), 35	Wilcoxon RS test
	Median [Range]	0.523 [-0.00, 2.00]	0.523 [-0.00, 2.00]	P=0.917

Item [unit]	Statistics Category	Group		Comparison between groups
		[1] The shallow anterior chamber phenotype (N=28)	[2] FECD (N=35)	
VA at last visit [logMAR]	Mean (SD), N	0.161 (0.426), 28	0.185 (0.346), 35	Wilcoxon RS test
	Median [Range]	0.046 [-0.08, 2.00]	0.097 [-0.08, 2.00]	P=0.041
Preoperative donor ECD [cells/mm²]	Mean (SD), N	2599.6 (171.7), 28	2670.9 (211.4), 35	Wilcoxon RS test
	Median [Range]	2621.5 [2020, 2901]	2663.0 [2050, 3083]	P=0.141
ECD at 12 months [cells/mm²]	Mean (SD), N	1206.1 (422.6), 28	1181.7 (514.8), 35	Wilcoxon RS test
	Median [Range]	1067.5 [363, 2257]	998.0 [578, 2519]	P=0.451
Loss of ECD at 12 months [%]	Mean (SD), N	53.90 (14.66), 28	56.51 (18.69), 35	Wilcoxon RS test
	Median [Range]	57.51 [17.7, 86.1]	64.37 [13.7, 77.9]	P=0.191
ECD at last visit [cells/mm²]	Mean (SD), N	929.0 (333.3), 28	1026.5 (466.2), 35	Wilcoxon RS test
	Median [Range]	844.0 [390, 1799]	823.0 [443, 1934]	P=0.870
Pre-DMEK CCT [µm]	Mean (SD), N	709.8 (97.0), 28	659.3 (89.6), 35	Wilcoxon RS test
	Median [Range]	691.0 [571, 937]	670.0 [501, 922]	P=0.041
CCT at last visit [µm]	Mean (SD), N	530.3 (65.9), 28	529.3 (52.0), 35	Wilcoxon RS test
	Median [Range]	528.5 [412, 767]	515.0 [441, 687]	P=0.972
Dark spots on specular microscopy	[1] No	16 (57.1%)	21 (60.0%)	Fisher's exact test
	[2] Yes	12 (42.9%)	14 (40.0%)	P=1.000
CME onset	[1] No	24 (85.7%)	32 (91.4%)	Fisher's exact test
	[2] Yes	4 (14.3%)	3 (8.6%)	P=0.690

Abbreviations: ACD, anterior chamber depth; CCT, central corneal thickness; CME, cystoid macular edema; DMEK, Descemet membrane endothelial keratoplasty; ECD, endothelial cell density; FECD, Fuchs endothelial corneal dystrophy; IOP, intraocular pressure; logMAR, logarithm of the minimum angle of resolution; RS, rank-sum; SD, standard deviation; VA, visual acuity.

S5 Table. Pre- and post-operative characteristics and measurements for PACD/ALI and shallow-AC groups.

Item [unit]	Statistics Category	Group		Comparison between groups
		[1] PACD/ALI (N=18)	[2] shallow-AC (N=10)	
Age [years]	Mean (SD), N	76.1 (5.6), 18	74.8 (6.3), 10	Wilcoxon RS test
	Median [Range]	77.0 [62, 86]	74.5 [65, 84]	P=0.486
Sex	[1] Male	2 (11.1%)	1 (10.0%)	Fisher's exact test
	[2] Female	16 (88.9%)	9 (90.0%)	P=1.000
Pre-cataract surgery ACD [mm]	Mean (SD), N	1.72 (0.24), 18	2.20 (0.43), 10	Wilcoxon RS test
	Median [Range]	1.71 [1.4, 2.1]	2.17 [1.7, 3.1]	P=0.005
Post-cataract surgery ACD [mm]	Mean (SD), N	3.61 (0.29), 18	3.75 (0.38), 10	Wilcoxon RS test
	Median [Range]	3.57 [3.1, 4.3]	3.89 [3.2, 4.2]	P=0.265
Acute primary angle closure	[1] No	12 (66.7%)	9 (90.0%)	Fisher's exact test
	[2] Yes	6 (33.3%)	1 (10.0%)	P=0.364
Rebubbling	[1] No	15 (83.3%)	7 (70.0%)	Fisher's exact test
	[2] Yes	3 (16.7%)	3 (30.0%)	P=0.634
Pre-DMEK IOP [mmHg]	Mean (SD), N	13.0 (3.8), 18	12.2 (3.0), 10	Wilcoxon RS test
	Median [Range]	12.0 [7, 21]	12.7 [7, 17]	P=0.718
Number of IOP-lowering medications before DMEK [counts]	[1] 0	15 (83.3%)	9 (90.0%)	Wilcoxon RS test
	[2] 1	0 (0.0%)	1 (10.0%)	P=0.581
	[3] 2	2 (11.1%)	0 (0.0%)	
	[4] 3	1 (5.6%)	0 (0.0%)	
Axial length [mm]	Mean (SD), N	22.53 (0.64), 18	22.44 (1.02), 10	Wilcoxon RS test
	Median [Range]	22.55 [21.3, 23.6]	22.46 [21.2, 24.5]	P=0.598

Item [unit]	Statistics Category	Group		Comparison between groups
		[1] PACD/ALI (N=18)	[2] shallow-AC (N=10)	
Pre-DMEK VA [logMAR]	Mean (SD), N	0.816 (0.583), 18	0.532 (0.417), 10	Wilcoxon RS test
	Median [Range]	0.523 [0.10, 2.00]	0.460 [-0.00, 1.22]	P=0.173
VA at last visit [logMAR]	Mean (SD), N	0.187 (0.482), 18	0.114 (0.320), 10	Wilcoxon RS test
	Median [Range]	0.046 [-0.08, 2.00]	0.046 [-0.08, 1.00]	P=0.660
Preoperative donor ECD [cells/mm²]	Mean (SD), N	2582.7 (203.7), 18	2630.1 (92.1), 10	Wilcoxon RS test
	Median [Range]	2612.5 [2020, 2901]	2632.0 [2506, 2743]	P=0.401
ECD at 12 months [cells/mm²]	Mean (SD), N	1123.9 (432.7), 18	1354.2 (379.9), 10	Wilcoxon RS test
	Median [Range]	1031.5 [363, 2043]	1300.0 [893, 2257]	P=0.089
Loss of ECD at 12 months [%]	Mean (SD), N	56.79 (14.96), 18	48.70 (13.24), 10	Wilcoxon RS test
	Median [Range]	60.59 [29.6, 86.1]	49.10 [17.7, 65.2]	P=0.133
ECD at last visit [cells/mm²]	Mean (SD), N	849.9 (296.6), 18	1063.3 (364.3), 10	Wilcoxon RS test
	Median [Range]	770.0 [390, 1672]	937.5 [720, 1799]	P=0.079
Pre-DMEK CCT [µm]	Mean (SD), N	741.3 (103.9), 18	653.1 (47.4), 10	Wilcoxon RS test
	Median [Range]	729.5 [571, 937]	652.5 [586, 745]	P=0.014
CCT at last visit [µm]	Mean (SD), N	537.5 (71.4), 18	517.4 (55.7), 10	Wilcoxon RS test
	Median [Range]	531.0 [440, 767]	512.5 [412, 585]	P=0.796
Dark spots on specular microscopy	[1] No	10 (55.6%)	6 (60.0%)	Fisher's exact test
	[2] Yes	8 (44.4%)	4 (40.0%)	P=1.000
CME onset	[1] No	15 (83.3%)	9 (90.0%)	Fisher's exact test
	[2] Yes	3 (16.7%)	1 (10.0%)	P=1.000

Abbreviations: ACD, anterior chamber depth; ALI, argon laser iridotomy; CCT, central corneal thickness; CME, cystoid macular edema; DMEK, Descemet membrane endothelial keratoplasty; ECD, endothelial cell density; IOP, intraocular pressure; logMAR, logarithm of the minimum angle of resolution; PACD, primary angle-closure disease; RS, rank-sum; SD, standard deviation; shallow-AC, shallow anterior chamber; VA, visual acuity.
