# Supplementary material for: Bullous keratopathy associated with a shallow anterior chamber: An anatomical risk phenotype for corneal endothelial decompensation
Source: PLoS One. 2026 Jul 7;21(7):e0353272. doi: 10.1371/journal.pone.0353272 (PMC13340822; doi:10.1371/journal.pone.0353272)
Supplement: Attachment — Submitted filename: Peer Review on BK associated with Shallow AC.pdf [file pone.0353272.s002.pdf]

Peer review on Bullous Keratopathy Associated with a Shallow Anterior Chamber: An Anatomical Risk Phenotype for Corneal Endothelial Decompensation

1) Spelling/Grammar mistakes:

- Keratopathy written incorrectly as Keratoparthy in pages (15,19,23)
- Data was shown, while better to use Data were shown (as plural)
- Analysis was shown to the latest, ideally would say Analysis were presented to the latest. (page 9)
- Eyes who were diagnosed, better to use eyes that were diagnosed (page 5)

2) Methods section:

-Explain how missing ACD pre-operative data were handled and whether those were equally distributed within the subgroups or were they more within a certain sub-group.

3) Discussion section:

Good interpretation of results and elaboration on the hypothetical cause of endothelial cell damage in eyes with shallow AC. However, as mentioned this is a hypothesis with small sample size and many confounding factors that would need to be controlled in order to reach a definite link between both Shallow AC and endothelial cell loss.
