# Supplementary material for: Bullous keratopathy associated with a shallow anterior chamber: An anatomical risk phenotype for corneal endothelial decompensation
Source: PLoS One. 2026 Jul 7;21(7):e0353272. doi: 10.1371/journal.pone.0353272 (PMC13340822; doi:10.1371/journal.pone.0353272)
Supplement: Attachment — Submitted filename: point-to-point.docx [file pone.0353272.s004.docx]

PONE-D-25-68588

Bullous Keratopathy Associated with a Shallow Anterior Chamber: An Anatomical Risk Phenotype for Corneal Endothelial Decompensation

PLOS One

Journal Requirements:

When submitting your revision, we need you to address these additional requirements.

1.Please ensure that your manuscript meets PLOS ONE's style requirements, including those for file naming. The PLOS ONE style templates can be found at

<https://journals.plos.org/plosone/s/file?id=wjVg/PLOSOne_formatting_sample_main_body.pdf> and

[https://journals.plos.org/plosone/s/file?id=ba62/PLOSOne_formatting_sample_title_authors_affiliations.pdf](https://track.editorialmanager.com/CL0/https:%2F%2Fjournals.plos.org%2Fplosone%2Fs%2Ffile%3Fid=ba62%2FPLOSOne_formatting_sample_title_authors_affiliations.pdf/1/010f019d2837122b-a8c28d8a-64d6-40c8-98d8-44c118849ffa-000000/UuMPfnoATIFQYOFSBgnvxrpLsJ7bPdgFFGf9gA-qngA=256)

Response: Thank you for your comment. We have revised the manuscript to conform to the PLOS ONE style requirements, including the title page, section headings, figure legends, and end-matter formatting. We have also checked the file naming format for the submission files in accordance with the journal guidelines.

2. We note that you have indicated that there are restrictions to data sharing for this study. PLOS only allows data to be available upon request if there are legal or ethical restrictions on sharing data publicly. For more information on unacceptable data access restrictions, please see <http://journals.plos.org/plosone/s/data-availability#loc-unacceptable-data-access-restrictions>.

Before we proceed with your manuscript, please address the following prompts:

a) If there are ethical or legal restrictions on sharing a de-identified data set, please explain them in detail (e.g., data contain potentially identifying or sensitive patient information, data are owned by a third-party organization, etc.) and who has imposed them (e.g., a Research Ethics Committee or Institutional Review Board, etc.). Please also provide contact information for a data access committee, ethics committee, or other institutional body to which data requests may be sent.

b) If there are no restrictions, please upload the minimal anonymized data set necessary to replicate your study findings to a stable, public repository and provide us with the relevant URLs, DOIs, or accession numbers. For a list of recommended repositories, please see

<https://journals.plos.org/plosone/s/recommended-repositories>. You also have the option of uploading the data as Supporting Information files, but we would recommend depositing data directly to a data repository if possible.

We will update your Data Availability statement on your behalf to reflect the information you provide.

Response: We thank the editor for this comment. There are no legal or ethical restrictions on sharing the minimal anonymized dataset necessary to replicate the study findings. We have uploaded the dataset as Supporting Information (S1 Dataset). The dataset was reviewed to remove potentially identifying information, including all specific dates, and an English-language version was prepared for submission. The Data Availability Statement in the manuscript reads: “All relevant data are within the manuscript and its Supporting Information files.”

Location: S1 Dataset, manuscript p34 Data availability statement

3. Your ethics statement should only appear in the Methods section of your manuscript. If your ethics statement is written in any section besides the Methods, please delete it from any other section.

Response: We thank the editor for this comment. The ethics statement was already fully described in the Methods section. We therefore removed the duplicate ethics statement from the end of the manuscript and retained it only in the Methods section.

Location: manuscript p5 Study design and ethics

4. Please upload a copy of Figure 3, to which you refer in your text on page 24. If the figure is no longer to be included as part of the submission please remove all reference to it within the text.

Response: We thank the editor for this comment. Figure 3 was included in the revised submission files, and all references to Figure 3 in the main text have been retained accordingly.

Location: Fig 3

5. We note that Figure 2 in your submission contain copyrighted images. All PLOS content is published under the Creative Commons Attribution License (CC BY 4.0), which means that the manuscript, images, and Supporting Information files will be freely available online, and any third party is permitted to access, download, copy, distribute, and use these materials in any way, even commercially, with proper attribution. For more information, see our copyright guidelines: <http://journals.plos.org/plosone/s/licenses-and-copyright>.

 We require you to either (1) present written permission from the copyright holder to publish these figures specifically under the CC BY 4.0 license, or (2) remove the figures from your submission:

 1. You may seek permission from the original copyright holder of Figure 2 to publish the content specifically under the CC BY 4.0 license.

 We recommend that you contact the original copyright holder with the Content Permission Form (<http://journals.plos.org/plosone/s/file?id=7c09/content-permission-form.pdf>) and the following text:

“I request permission for the open-access journal PLOS ONE to publish XXX under the Creative Commons Attribution License (CCAL) CC BY 4.0 (<http://creativecommons.org/licenses/by/4.0/>). Please be aware that this license allows unrestricted use and distribution, even commercially, by third parties. Please reply and provide explicit written permission to publish XXX under a CC BY license and complete the attached form.”

 Please upload the completed Content Permission Form or other proof of granted permissions as an "Other" file with your submission.

 In the figure caption of the copyrighted figure, please include the following text: “Reprinted from [ref] under a CC BY license, with permission from [name of publisher], original copyright [original copyright year].”

 2. If you are unable to obtain permission from the original copyright holder to publish these figures under the CC BY 4.0 license or if the copyright holder’s requirements are incompatible with the CC BY 4.0 license, please either i) remove the figure or ii) supply a replacement figure that complies with the CC BY 4.0 license. Please check copyright information on all replacement figures and update the figure caption with source information. If applicable, please specify in the figure caption text when a figure is similar but not identical to the original image and is therefore for illustrative purposes only.

Response: We thank the editor for this comment. Figure 2 is an original figure created by the authors. Although an earlier version of the manuscript was posted on Research Square, the authors retained the copyright to the preprint and all included figures, while Research Square holds only a non-exclusive license to distribute the content. No copyright transfer to any publisher has occurred. Therefore, the authors retain the right to publish Figure 2 under the CC BY 4.0 license required by PLOS ONE, and no additional third-party permission is required.

6. Thank you for providing your underlying data as Supporting Information.

We note that the data set contains text or data that is not in English. Please note that PLOS is an English-language publisher, so we require data sets to be provided in English as well. Please upload an English-language version of your data set.

This will also allow us to determine if your data follows PLOS standards per our Data Availability policy here: [https://journals.plos.org/plosone/s/data-availability](https://track.editorialmanager.com/CL0/https:%2F%2Fjournals.plos.org%2Fplosone%2Fs%2Fdata-availability/1/010f019d2837122b-a8c28d8a-64d6-40c8-98d8-44c118849ffa-000000/2Jm79mjzSKR5Z4oRuX71SITvAtysDLeH8qyTmfLZnlU=256).

Response: We thank the editor for this comment. We revised the dataset and uploaded an English-language version as Supporting Information.

Location: S1 Dataset

7. We note that there is identifying data in the Supporting Information file “dataset.pdf”. Due to the inclusion of these potentially identifying data, we have removed this file from your file inventory. Prior to sharing human research participant data, authors should consult with an ethics committee to ensure data are shared in accordance with participant consent and all applicable local laws.

Data sharing should never compromise participant privacy. It is therefore not appropriate to publicly share personally identifiable data on human research participants. The following are examples of data that should not be shared:

-Name, initials, physical address

-Ages more specific than whole numbers

-Internet protocol (IP) address

-Specific dates (birth dates, death dates, examination dates, etc.)

-Contact information such as phone number or email address

-Location data

-ID numbers that seem specific (long numbers, include initials, titled “Hospital ID”) rather than random (small numbers in numerical order)

Data that are not directly identifying may also be inappropriate to share, as in combination they can become identifying. For example, data collected from a small group of participants, vulnerable populations, or private groups should not be shared if they involve indirect identifiers (such as sex, ethnicity, location, etc.) that may risk the identification of study participants.

Additional guidance on preparing raw data for publication can be found in our Data Policy (<https://journals.plos.org/plosone/s/data-availability#loc-human-research-participant-data-and-other-sensitive-data>) and in the following article: <http://www.bmj.com/content/340/bmj.c181.long>.

Please remove or anonymize all personal information (<specific identifying information in file to be removed>), ensure that the data shared are in accordance with participant consent, and re-upload a fully anonymized data set. Please note that spreadsheet columns with personal information must be removed and not hidden as all hidden columns will appear in the published file.

Response: We thank the editor for this important comment. We revised the Supporting Information dataset to remove potentially identifying information, including all specific dates, and prepared an English-language version for re-upload. Age is presented only in whole numbers, and no direct personal identifiers are included. We also confirmed that columns containing personal information were removed rather than hidden. The revised dataset has been anonymized in accordance with participant privacy requirements, institutional regulations, and the journal’s data-sharing policy.

Location: S1 Dataset

8. If the reviewer comments include a recommendation to cite specific previously published works, please review and evaluate these publications to determine whether they are relevant and should be cited. There is no requirement to cite these works unless the editor has indicated otherwise.

Response: We thank the editor for this comment. We reviewed the publications recommended by the reviewers, found them relevant to the present study, and incorporated the appropriate citations into the revised Discussion.

Location: manuscript p27-28 Line 340-347, 361-368

9. Please review your reference list to ensure that it is complete and correct. If you have cited papers that have been retracted, please include the rationale for doing so in the manuscript text, or remove these references and replace them with relevant current references. Any changes to the reference list should be mentioned in the rebuttal letter that accompanies your revised manuscript. If you need to cite a retracted article, indicate the article’s retracted status in the References list and also include a citation and full reference for the retraction notice.

Response: We thank the editor for this comment. We carefully reviewed the reference list for completeness and accuracy and revised it as needed in the updated manuscript. We also confirmed that no retracted articles are cited.

Location: manuscript p31-33

Additional Editor Comments:

- Please address particularly the limitations inherent to small sample size.

Response: We thank the editor for this important comment. We revised the Discussion and Limitations sections to more clearly acknowledge the limitations related to the small sample size, particularly in the PAC/Unknown subgroup. Specifically, we emphasized the limited sample size, the relatively short follow-up, the possibility of limited statistical power in some analyses, and the exploratory nature of the findings, and we clarified that larger studies with longer follow-up are needed.

Location: manuscript p30 Limitations

[Note: HTML markup is below. Please do not edit.]

Reviewers' comments:
Reviewer's Responses to Questions

**Comments to the Author**

5. Review Comments to the Author
Please use the space provided to explain your answers to the questions above. You may also include additional comments for the author, including concerns about dual publication, research ethics, or publication ethics. (Please upload your review as an attachment if it exceeds 20,000 characters)

Reviewer #1: The work highlights an important and often under-recognized phenotype, and the imaging documentation is excellent. To further strengthen the study and align it with current understanding of postoperative endothelial and stromal behavior, I would encourage you to address the following points.

-Please discuss the recent literature that it has demonstrated that PSR are not merely descriptive findings but structural biomarkers of stromal overhydration and lamellar micro-distortion. In particular, preoperative PSR correlate with slower visual recovery and postoperative PSR strongly predict rebubbling and graft instability (Ventura et al., Cornea 2024). Including this discussion will contextualize your observations and provide a more mechanistic interpretation of stromal morphology in your cohort.

Response: Thank you for this insightful comment. We have expanded the Discussion to incorporate the recent literature on posterior stromal ripples (PSR). Specifically, we clarified that PSR are not merely descriptive findings on anterior segment optical coherence tomography, but structural biomarkers of stromal overhydration and lamellar micro-distortion after DMEK. We also added discussion of the reported associations between preoperative PSR and slower visual recovery, as well as between postoperative PSR and rebubbling/graft instability. In addition, we related these findings to the posterior stromal irregularity observed in our illustrative case, while acknowledging that PSR were not systematically assessed in the present cohort.

Location: manuscript p27-28 Line 340-347, 361-368

-Discuss the role of subclinical inflammation. AS-OCT–based studies have shown that subclinical anterior chamber inflammation is almost universally present after DMEK, and higher postoperative inflammatory load (measured by ARI index and hyperreflective dots) is linked to the development of postoperative PSR and rebubbling (Vaccaro et al., Ophthalmology Science 2026). Incorporating this concept can help explain why some eyes demonstrate unexpected endothelial stress or postoperative behavior despite uneventful surgery.

Response: Thank you for this important comment. We have expanded the Discussion to address the role of subclinical postoperative inflammation after DMEK. Specifically, we added discussion of AS-OCT–based evidence showing that higher postoperative inflammatory load, reflected by the ARI index and hyperreflective dots, is associated with PSR and with postoperative rebubbling/graft instability. We also noted that this concept may help explain variable postoperative behavior despite apparently uneventful surgery.

Location: manuscript p27-28 Line 340-347, 361-368

-clarify the mechanistic link between anatomy and endothelial vulnerability. Your hypothesis of a shallow-anterior-chamber risk phenotype is intriguing, but discussing how crowding, micro-shear forces, and low-grade inflammation may contribute to chronic endothelial stress would strengthen the biological plausibility of the proposed mechanism.

Response: Thank you for this helpful suggestion. We have expanded the Discussion to clarify the potential mechanistic link between shallow anterior chamber anatomy and endothelial vulnerability. Specifically, we added discussion of anterior segment crowding, altered aqueous flow and micro-shear forces, intermittent IOP elevation, episodic iridocorneal contact, and low-grade inflammation as possible contributors to chronic endothelial stress and endothelial decompensation in this anatomical setting.

Location: manuscript p29-30 Line 388-395

-address statistical and subgroup limitations, several conclusions rely on small subgroups and unadjusted analyses. A more cautious interpretation, along with acknowledgment of potential confounders and selection bias, will help support the credibility of your results.

Response: Thank you for this important comment. We have revised the Limitations section to provide a more cautious interpretation of our findings. Specifically, we clarified that the PAC/Unknown subgroup was small and had relatively short follow-up, that most analyses were univariable and unadjusted, and that potential confounding and selection bias inherent to the retrospective design could not be excluded. We also emphasized that our findings should be interpreted as exploratory and hypothesis-generating rather than causal.

Location: manuscript p30 Limitations

Reviewer #2: Interesting area to study as it has been previously proven that APAC or ALI contributes to endothelial cell loss and future corneal decompensation. However, anatomical risk factor by itself without any superadded factor is an interesting area.
However, the number of patients involved in the desired area is quite small and cannot base a conclusion completely out of it.
Would be nice to elaborate on the study on a larger scale to review the impact of a shallow AC on the ECD.

Response: Thank you for this important comment. We agree that the number of eyes in the PAC/Unknown subgroup was limited and that this subgroup alone is insufficient to support definitive conclusions. We have therefore revised the manuscript to interpret these findings more cautiously and to emphasize that they should be regarded as exploratory and hypothesis-generating. We also added/strengthened the statement that larger studies will be necessary to better clarify the impact of shallow anterior chamber anatomy on endothelial vulnerability and corneal decompensation.

Location: manuscript p30 Limitations, manuscript p2 Abstract, manuscript p27-31 Discussion

[NOTE: If reviewer comments were submitted as an attachment file, they will be attached to this email and accessible via the submission site. Please log into your account, locate the manuscript record, and check for the action link "View Attachments". If this link does not appear, there are no attachment files.]

Peer review on Bullous Keratopathy Associated with a Shallow Anterior Chamber: An Anatomical Risk Phenotype for Corneal Endothelial Decompensation

1) Spelling/Grammar mistakes:

- Keratopathy written incorrectly as Keratoparthy in pages ( 15,19,23)

- Data was shown, while better to use Data were shown ( as plural )

- Analysis was shown to the latest, ideally would say Analysis were presented to the latest. ( page 9 )

- Eyes who were diagnosed, better to use eyes that were diagnosed ( page 5 )

Response: Thank you for these careful comments. We have corrected the spelling of “keratopathy” throughout the manuscript, revised the relevant sentence(s) to improve subject–verb agreement and phrasing, including the expressions related to “data” and “analysis,” and changed “eyes who were diagnosed” to “eyes that were diagnosed.”

Location: manuscript Line 238, 263, 270, 218, 107

2) Methods section:

-Explain how missing ACD pre-operative data were handled and whether those were equally distributed within the subgroups or were they more within a certain sub-group.

Response: Thank you for this helpful comment. We have clarified in the Methods section how missing pre-cataract surgery ACD data were handled. Specifically, pre-cataract surgery ACD was analyzed on an available-case basis, and no imputation was performed for missing values. We also added that missing pre-cataract surgery ACD data were not equally distributed across subgroups; all ABK and PBK eyes lacked these measurements, whereas all PAC/Unknown eyes had available measurements.

Location: manuscript p7-8 Statistical analysis

3) Discussion section:

Good interpretation of results and elaboration on the hypothetical cause of endothelial cell damage in eyes with shallow AC. However, as mentioned this is a hypothesis with small sample size and many confounding factors that would need to be controlled in order to reach a definite link between both Shallow AC and endothelial cell loss.

Response: Thank you for this important comment. We agree that the proposed link between shallow anterior chamber anatomy and endothelial cell loss remains hypothetical and should be interpreted cautiously, given the small subgroup size and the presence of potential confounding factors. We therefore revised the Discussion and Limitations sections to emphasize that most analyses were univariable, that potential confounding and selection bias could not be excluded, and that our findings should be regarded as exploratory and hypothesis-generating rather than definitive or causal.

Location: manuscript p30 Limitations, manuscript p2 Abstract, manuscript p27-31 Discussion
