# Supplementary material for: Bullous keratopathy associated with a shallow anterior chamber: An anatomical risk phenotype for corneal endothelial decompensation
Source: PLoS One. 2026 Jul 7;21(7):e0353272. doi: 10.1371/journal.pone.0353272 (PMC13340822; doi:10.1371/journal.pone.0353272)
Supplement: Attachment — Submitted filename: Point_to_point_DMEK_for_shallow_AC.docx [file pone.0353272.s005.docx]

Response to Academic Editor

Manuscript ID: PONE-D-25-68588R1

Title: Bullous keratopathy associated with a shallow anterior chamber: an anatomical risk phenotype for corneal endothelial decompensation

Dear Dr. Lalgudi,

We sincerely thank you and the reviewers for the careful evaluation of our manuscript and for the constructive comments. We have revised the manuscript accordingly to improve the clarity, reproducibility, and validity of the study. All changes have been incorporated into the revised manuscript and are indicated in the tracked-changes version.

Below, we provide a point-by-point response to each comment.

Comment 1

Editor’s comment:

Firstly, to improve the repeatability of the methodology, we need to have a clear explanation on how PAC was diagnosed. Was it just based on ACD, or based on gonioscopic findings as well? There seems to be no description of that. If gonioscopy was performed, was there any variation on the grade of the angle, including presence or absence and extent of PAS in the patients studied? Was ASOCT performed to analyze the trabecular iris angle (TIA)? And, please describe 'dark spots' on specular microscopy in the methodology.

Response:

Thank you for this important comment. We agree that the previous terminology may have been misleading. In the original manuscript, we used the terms “PAC/ALI” and “PAC/Unknown.” However, because the former “PAC/Unknown” group did not have sufficient documentation to support a strict diagnosis of PACD based on uniform gonioscopic criteria, we revised the terminology throughout the manuscript. Specifically, we renamed this group as the “shallow anterior chamber (shallow-AC) group” and clarified that this classification was intended to represent a shallow anterior chamber phenotype rather than a definitive diagnosis of PACD.

We also revised the Methods section to clarify how PACD was diagnosed. The PACD/ALI group was defined based on medical records documenting a history of argon laser iridotomy performed for a clinically diagnosed PACD-spectrum condition, supported when available by gonioscopic angle closure and/or a clinical diagnosis by glaucoma or corneal specialists. We further clarified that, because of the retrospective nature of the study, standardized gonioscopic grading and the extent of peripheral anterior synechiae were not consistently available and were therefore not included as analytic variables.

In addition, we clarified that AS-OCT was used to measure ACD and CCT, but angle parameters, including the trabecular iris angle, were not uniformly measured and were not used for group classification or statistical analyses. Finally, we added a definition of dark spots on specular microscopy and clarified that dark spots alone were not used as independent diagnostic criteria for FECD.

Location:
Abstract; Methods, Patients and data collection; Methods, Ophthalmic examinations; Results; Discussion; Figure legends; Supporting Information.

Comment 2

Editor’s comment:

As cornea specialists, when we see patients with BK without evidence of FECD (clinically/on specular of affected/fellow eye), the following are the differentials. Number 1 is cataract surgery or phaco energy induced endothelial loss or PBK. Especially in eyes with shallow ACD or with denser cataracts, the impact of phaco energy on the endothelium is significant. IOL position is a significant concern as well. Sulcus IOLs or UGH concerns or ACIOLs are known to increase the risk of corneal decompensation. Other aspects to rule out are a viral masquerade. Patients with a HSV or CMV endotheliitis in the past can also present with endothelial dysfunction and can improve with oral antivirals and topical steroids. While trying to describe a group of BK eyes with PAC without APAC or ALI, one of the major concerns is the fact that the authors seem to be completely ignoring the role and impact of cataract surgery and other above mentioned factors in these patients. The following parameters need to be added to the article. a) What was the timeline of the cataract surgery before presenting with corneal edema? b) Cataract surgery parameters including CDE (cumulative dissipated energy), IOL used and position of the IOL, c) How long prior to Cataract surgery was LPI performed? d) Pre-cataract surgery ECD on specular microscopy. e) Was any aqueous tap performed for ruling out HSV/CMV IgG/IgM PCR in BK patients with no evidence of FECD?

Response:

We thank the Editor for this important comment. We agree that cataract surgery-related endothelial injury, IOL-related factors, and viral endotheliitis are important differential considerations when evaluating bullous keratopathy without evidence of FECD. To address this concern, we reviewed additional cataract surgery-related variables in the shallow anterior chamber phenotype group and added these data to the Results section under “Patient characteristics” and to S2 Table.

Specifically, we added information on the timing of cataract surgery relative to corneal edema confirmation, cumulative dissipated energy, IOL type and position, the interval from ALI to cataract surgery, pre-cataract surgery ECD, and clinical findings and aqueous humor testing related to possible viral endotheliitis. Among the 28 eyes in the shallow anterior chamber phenotype group, 22 eyes underwent cataract surgery as part of a planned staged surgical approach before DMEK after corneal endothelial decompensation or BK had already been recognized. Cumulative dissipated energy was not available in the medical records. Among eyes with available IOL position data, all IOLs were placed in the bag, and no sulcus IOL or anterior chamber IOL was documented. The interval from ALI to cataract surgery and pre-cataract surgery ECD are summarized in S2 Table. Clinical findings suggestive of viral endotheliitis were noted in one eye, and aqueous humor testing, when performed, was negative.

These additions clarify that cataract surgery-related factors, IOL position, prior ALI, pre-cataract endothelial status, and possible viral masquerade were specifically reviewed in response to the Editor’s concern.

Although cumulative dissipated energy was not available because of the retrospective nature of the study, the newly added data indicate that most cataract surgeries in this subgroup were performed as planned staged procedures after endothelial decompensation or BK had already been recognized, and that no sulcus IOL or anterior chamber IOL was documented among evaluable eyes.

Location:
Results, Patient characteristics; Supporting Information, S2 Table.

Comment 3

Editor’s comment:

In the results section, what does 'baseline' visit represent? Is it prior to cataract surgery or prior to DMEK surgery?

Response:

Thank you for this important comment. To avoid ambiguity, we revised the Methods section and removed the term “baseline” from the relevant descriptions. We now specify that VA, CCT, IOP, the number of IOP-lowering medications, axial length, and preoperative donor ECD were recorded before DMEK. ACD was evaluated separately as pre-cataract surgery ACD and post-cataract surgery ACD. We also clarified that post-cataract surgery ACD was measured at the pre-DMEK assessment.

Location: Methods, Ophthalmic examinations; Results, Patient characteristics; Supporting Information, S4 and S5 Tables.

Comment 4

Editor’s comment:

Table 1 is confusing. After the row on 'Breakdown of BK', please create a separate table with new headers to make it easy for readers.

Response:

Thank you for this helpful suggestion. We revised Table 1 to improve readability. Specifically, we separated the original “Breakdown of BK” section from the main patient demographics and clinical characteristics table and created a separate table with new headers for BK cases. The main patient characteristics are now presented in Table 1A, and the breakdown of BK cases is presented separately in Table 1B. We also revised the corresponding text citation in the Results section.

Location: Results, Patient characteristics; Tables 1A and 1B.

Comment 5

Editor’s comment:

In table 1, 19 patients are mentioned to have Laser iridotomy. However, your PAC group with ALI is only 18. Why is this discrepancy?

Response:

Thank you for pointing this out. This discrepancy was due to a transcription error in the previous version of Table 1. We reviewed the dataset and confirmed that 18 eyes had a history of laser iridotomy, which is consistent with the number of eyes in the PACD/ALI group. We corrected the value in Table 1A accordingly.

Location: Table 1A.

Comment 6

Editor’s comment:

Line 315 mentions that PEX had deeper chambers than those with PAC eyes. However, Table 2 mentions PEX ACD mean as 2.09mm versus PAC group with 2.20 mm mean. Please fix this discrepancy.

Response:

We thank the editor for pointing out this discrepancy. We agree that the original statement was imprecise. In Table 2, the mean pre-cataract surgery ACD was 2.09 mm in the PEX subgroup and 2.20 mm in the shallow-AC subgroup; therefore, it was inappropriate to state that PEX was associated with deeper chambers in this context. We deleted the sentence “Conversely, FECD and PEX were associated with deeper chambers” and revised the Discussion to avoid this misleading interpretation. The revised sentence now reads: “Together, these findings suggest that eyes with the shallow anterior chamber phenotype may have anterior segment characteristics that differ from those of eyes with FECD.”

Location: Discussion, first paragraph.

Comment 7

Editor’s comment:

The following article has also previously described CECD cell loss in PACS eyes without ALI or angle closure attacks. Kusumi Y, Yamamoto M, Fukui M, Yamada M. Morphological Features in Eyes with Prominent Corneal Endothelial Cell Loss Associated with Primary Angle-Closure Disease. J Clin Med. 2025 Jul 29;14(15):5364. doi: 10.3390/jcm14155364. PMID: 40806986; PMCID: PMC12346976. This article points out the role of keratometry or radius of curvature in CECD loss in PACS eyes. Are you able to include corneal curvature in your patients in the study as well? Please also include this study in the discussion section.

Response:
We appreciate the Editor’s important suggestion. We agree that the study by Kusumi et al. is highly relevant, because it reported prominent corneal endothelial cell density loss in eyes with primary angle-closure disease, including primary angle-closure suspect eyes without a history of acute angle-closure attacks or laser iridotomy, and suggested a potential role of corneal curvature radius in endothelial vulnerability. We have incorporated this study into the Discussion.

Unfortunately, keratometric data and corneal curvature parameters were not consistently available because of the retrospective nature of this study and the long study period. Therefore, we were unable to perform a reliable corneal curvature analysis in the present cohort.

Location: Discussion.

Additional revisions

In addition to the changes made in response to the editor’s comments, we carefully reviewed the manuscript for clarity, consistency, and typographical errors.

Sincerely,
Takahiko Hayashi
On behalf of all authors
