# Supplementary material for: Bullous keratopathy associated with a shallow anterior chamber: An anatomical risk phenotype for corneal endothelial decompensation
Source: PLoS One. 2026 Jul 7;21(7):e0353272. doi: 10.1371/journal.pone.0353272 (PMC13340822; doi:10.1371/journal.pone.0353272)
Supplement: Peer review history

# Peer review: Decision Letter 0

**DOI:** 10.1371/journal.pone.0353272.r001

**Authors:** Nader Hussien Lotfy Bayoumi

25 Mar 2026

PONE-D-25-68588

Bullous Keratopathy Associated with a Shallow Anterior Chamber: An Anatomical Risk Phenotype for Corneal Endothelial Decompensation

PLOS One

Dear Dr. Hayashi,

Thank you for submitting your manuscript to PLOS ONE. After careful consideration, we feel that it has merit but does not fully meet PLOS ONE’s publication criteria as it currently stands. Therefore, we invite you to submit a revised version of the manuscript that addresses the points raised during the review process.

Please submit your revised manuscript by May 09 2026 11:59PM. If you will need more time than this to complete your revisions, please reply to this message or contact the journal office at plosone@plos.org. When you're ready to submit your revision, log on to https://www.editorialmanager.com/pone/ and select the 'Submissions Needing Revision' folder to locate your manuscript file.

Please include the following items when submitting your revised manuscript:

- A letter that responds to each point raised by the academic editor and reviewer(s). You should upload this letter as a separate file labeled 'Response to Reviewers'.
- A marked-up copy of your manuscript that highlights changes made to the original version. You should upload this as a separate file labeled 'Revised Manuscript with Track Changes'.
- An unmarked version of your revised paper without tracked changes. You should upload this as a separate file labeled 'Manuscript'.

If you would like to make changes to your financial disclosure, please include your updated statement in your cover letter. Guidelines for resubmitting your figure files are available below the reviewer comments at the end of this letter.

If applicable, we recommend that you deposit your laboratory protocols in protocols.io to enhance the reproducibility of your results. Protocols.io assigns your protocol its own identifier (DOI) so that it can be cited independently in the future. For instructions see: https://journals.plos.org/plosone/s/submission-guidelines#loc-laboratory-protocols. Additionally, PLOS ONE offers an option for publishing peer-reviewed Lab Protocol articles, which describe protocols hosted on protocols.io. Read more information on sharing protocols at https://plos.org/protocols?utm_medium=editorial-email&utm_source=authorletters&utm_campaign=protocols.

As the corresponding author, your ORCID iD is verified in the submission system and will appear in the published article. PLOS supports the use of ORCID, and we encourage all coauthors to register for an ORCID iD and use it as well. Please encourage your coauthors to verify their ORCID iD within the submission system before final acceptance, as unverified ORCID iDs will not appear in the published article. *Only*  the individual author can complete the verification step; PLOS staff *cannot*  verify ORCID iDs on behalf of authors.

We look forward to receiving your revised manuscript.

Kind regards,

Nader Hussien Lotfy Bayoumi, M.D., FRCS (Glasgow)

Academic Editor

PLOS One

Journal Requirements:

When submitting your revision, we need you to address these additional requirements.

1.Please ensure that your manuscript meets PLOS ONE's style requirements, including those for file naming. The PLOS ONE style templates can be found at

https://journals.plos.org/plosone/s/file?id=wjVg/PLOSOne_formatting_sample_main_body.pdf and

https://journals.plos.org/plosone/s/file?id=ba62/PLOSOne_formatting_sample_title_authors_affiliations.pdf

2. We note that you have indicated that there are restrictions to data sharing for this study. PLOS only allows data to be available upon request if there are legal or ethical restrictions on sharing data publicly. For more information on unacceptable data access restrictions, please see http://journals.plos.org/plosone/s/data-availability#loc-unacceptable-data-access-restrictions.

Before we proceed with your manuscript, please address the following prompts:

a) If there are ethical or legal restrictions on sharing a de-identified data set, please explain them in detail (e.g., data contain potentially identifying or sensitive patient information, data are owned by a third-party organization, etc.) and who has imposed them (e.g., a Research Ethics Committee or Institutional Review Board, etc.). Please also provide contact information for a data access committee, ethics committee, or other institutional body to which data requests may be sent.

b) If there are no restrictions, please upload the minimal anonymized data set necessary to replicate your study findings to a stable, public repository and provide us with the relevant URLs, DOIs, or accession numbers. For a list of recommended repositories, please see

https://journals.plos.org/plosone/s/recommended-repositories. You also have the option of uploading the data as Supporting Information files, but we would recommend depositing data directly to a data repository if possible.

We will update your Data Availability statement on your behalf to reflect the information you provide.

3. Your ethics statement should only appear in the Methods section of your manuscript. If your ethics statement is written in any section besides the Methods, please delete it from any other section.

4. Please upload a copy of Figure 3, to which you refer in your text on page 24. If the figure is no longer to be included as part of the submission please remove all reference to it within the text.

5. We note that Figure 2 in your submission contain copyrighted images. All PLOS content is published under the Creative Commons Attribution License (CC BY 4.0), which means that the manuscript, images, and Supporting Information files will be freely available online, and any third party is permitted to access, download, copy, distribute, and use these materials in any way, even commercially, with proper attribution. For more information, see our copyright guidelines: http://journals.plos.org/plosone/s/licenses-and-copyright.

We require you to either (1) present written permission from the copyright holder to publish these figures specifically under the CC BY 4.0 license, or (2) remove the figures from your submission:

1. You may seek permission from the original copyright holder of Figure 2 to publish the content specifically under the CC BY 4.0 license.

We recommend that you contact the original copyright holder with the Content Permission Form (http://journals.plos.org/plosone/s/file?id=7c09/content-permission-form.pdf) and the following text:

“I request permission for the open-access journal PLOS ONE to publish XXX under the Creative Commons Attribution License (CCAL) CC BY 4.0 (http://creativecommons.org/licenses/by/4.0/). Please be aware that this license allows unrestricted use and distribution, even commercially, by third parties. Please reply and provide explicit written permission to publish XXX under a CC BY license and complete the attached form.”

Please upload the completed Content Permission Form or other proof of granted permissions as an "Other" file with your submission.

In the figure caption of the copyrighted figure, please include the following text: “Reprinted from [ref] under a CC BY license, with permission from [name of publisher], original copyright [original copyright year].”

2. If you are unable to obtain permission from the original copyright holder to publish these figures under the CC BY 4.0 license or if the copyright holder’s requirements are incompatible with the CC BY 4.0 license, please either i) remove the figure or ii) supply a replacement figure that complies with the CC BY 4.0 license. Please check copyright information on all replacement figures and update the figure caption with source information. If applicable, please specify in the figure caption text when a figure is similar but not identical to the original image and is therefore for illustrative purposes only.

6. Thank you for providing your underlying data as Supporting Information.

We note that the data set contains text or data that is not in English. Please note that PLOS is an English-language publisher, so we require data sets to be provided in English as well. Please upload an English-language version of your data set.

This will also allow us to determine if your data follows PLOS standards per our Data Availability policy here: https://journals.plos.org/plosone/s/data-availability.

7. We note that there is identifying data in the Supporting Information file “dataset.pdf”. Due to the inclusion of these potentially identifying data, we have removed this file from your file inventory. Prior to sharing human research participant data, authors should consult with an ethics committee to ensure data are shared in accordance with participant consent and all applicable local laws.

Data sharing should never compromise participant privacy. It is therefore not appropriate to publicly share personally identifiable data on human research participants. The following are examples of data that should not be shared:

-Name, initials, physical address

-Ages more specific than whole numbers

-Internet protocol (IP) address

-Specific dates (birth dates, death dates, examination dates, etc.)

-Contact information such as phone number or email address

-Location data

-ID numbers that seem specific (long numbers, include initials, titled “Hospital ID”) rather than random (small numbers in numerical order)

Data that are not directly identifying may also be inappropriate to share, as in combination they can become identifying. For example, data collected from a small group of participants, vulnerable populations, or private groups should not be shared if they involve indirect identifiers (such as sex, ethnicity, location, etc.) that may risk the identification of study participants.

Additional guidance on preparing raw data for publication can be found in our Data Policy (https://journals.plos.org/plosone/s/data-availability#loc-human-research-participant-data-and-other-sensitive-data) and in the following article: http://www.bmj.com/content/340/bmj.c181.long.

Please remove or anonymize all personal information (<specific identifying information in file to be removed>), ensure that the data shared are in accordance with participant consent, and re-upload a fully anonymized data set. Please note that spreadsheet columns with personal information must be removed and not hidden as all hidden columns will appear in the published file.

8. If the reviewer comments include a recommendation to cite specific previously published works, please review and evaluate these publications to determine whether they are relevant and should be cited. There is no requirement to cite these works unless the editor has indicated otherwise.

9. Please review your reference list to ensure that it is complete and correct. If you have cited papers that have been retracted, please include the rationale for doing so in the manuscript text, or remove these references and replace them with relevant current references. Any changes to the reference list should be mentioned in the rebuttal letter that accompanies your revised manuscript. If you need to cite a retracted article, indicate the article’s retracted status in the References list and also include a citation and full reference for the retraction notice.

Additional Editor Comments:

- Please address particularly the limitations inherent to small sample size.

[Note: HTML markup is below. Please do not edit.]

Reviewers' comments:

Reviewer's Responses to Questions

**Comments to the Author**

1. Is the manuscript technically sound, and do the data support the conclusions?

The manuscript must describe a technically sound piece of scientific research with data that supports the conclusions. Experiments must have been conducted rigorously, with appropriate controls, replication, and sample sizes. The conclusions must be drawn appropriately based on the data presented.

Reviewer #1: Yes

Reviewer #2: Yes

**********

2. Has the statistical analysis been performed appropriately and rigorously?

Reviewer #1: Yes

Reviewer #2: Yes

**********

3. Have the authors made all data underlying the findings in their manuscript fully available?

The PLOS Data policy requires authors to make all data underlying the findings described in their manuscript fully available without restriction, with rare exception (please refer to the Data Availability Statement in the manuscript PDF file). The data should be provided as part of the manuscript or its supporting information, or deposited to a public repository. For example, in addition to summary statistics, the data points behind means, medians and variance measures should be available. If there are restrictions on publicly sharing data—e.g. participant privacy or use of data from a third party—those must be specified.

Reviewer #1: Yes

Reviewer #2: Yes

**********

4. Is the manuscript presented in an intelligible fashion and written in standard English?

PLOS ONE does not copyedit accepted manuscripts, so the language in submitted articles must be clear, correct, and unambiguous. Any typographical or grammatical errors should be corrected at revision, so please note any specific errors here.

Reviewer #1: Yes

Reviewer #2: Yes

**********

5. Review Comments to the Author

Please use the space provided to explain your answers to the questions above. You may also include additional comments for the author, including concerns about dual publication, research ethics, or publication ethics. (Please upload your review as an attachment if it exceeds 20,000 characters)

Reviewer #1: The work highlights an important and often under-recognized phenotype, and the imaging documentation is excellent. To further strengthen the study and align it with current understanding of postoperative endothelial and stromal behavior, I would encourage you to address the following points.

-Please discuss the recent literature that it has demonstrated that PSR are not merely descriptive findings but structural biomarkers of stromal overhydration and lamellar micro-distortion. In particular, preoperative PSR correlate with slower visual recovery and postoperative PSR strongly predict rebubbling and graft instability (Ventura et al., Cornea 2024). Including this discussion will contextualize your observations and provide a more mechanistic interpretation of stromal morphology in your cohort.

-Discuss the role of subclinical inflammation. AS-OCT–based studies have shown that subclinical anterior chamber inflammation is almost universally present after DMEK, and higher postoperative inflammatory load (measured by ARI index and hyperreflective dots) is linked to the development of postoperative PSR and rebubbling (Vaccaro et al., Ophthalmology Science 2026). Incorporating this concept can help explain why some eyes demonstrate unexpected endothelial stress or postoperative behavior despite uneventful surgery.

-clarify the mechanistic link between anatomy and endothelial vulnerability. Your hypothesis of a shallow-anterior-chamber risk phenotype is intriguing, but discussing how crowding, micro-shear forces, and low-grade inflammation may contribute to chronic endothelial stress would strengthen the biological plausibility of the proposed mechanism.

-address statistical and subgroup limitations, several conclusions rely on small subgroups and unadjusted analyses. A more cautious interpretation, along with acknowledgment of potential confounders and selection bias, will help support the credibility of your results.

Reviewer #2: Interesting area to study as it has been previously proven that APAC or ALI contributes to endothelial cell loss and future corneal decompensation. However, anatomical risk factor by itself without any superadded factor is an interesting area.

However, the number of patients involved in the desired area is quite small and cannot base a conclusion completely out of it.

Would be nice to elaborate on the study on a larger scale to review the impact of a shallow AC on the ECD.

**********

6. PLOS authors have the option to publish the peer review history of their article (what does this mean?). If published, this will include your full peer review and any attached files.

If you choose “no”, your identity will remain anonymous but your review may still be made public.

**Do you want your identity to be public for this peer review?**  For information about this choice, including consent withdrawal, please see our Privacy Policy.

Reviewer #1: No

Reviewer #2: **Yes:** Karim Amr Mostafa Elsayed

**********

[NOTE: If reviewer comments were submitted as an attachment file, they will be attached to this email and accessible via the submission site. Please log into your account, locate the manuscript record, and check for the action link "View Attachments". If this link does not appear, there are no attachment files.]

To ensure your figures meet our technical requirements, please review our figure guidelines: https://journals.plos.org/plosone/s/figures

You may also use PLOS’s free figure tool, NAAS, to help you prepare publication quality figures: https://journals.plos.org/plosone/s/figures#loc-tools-for-figure-preparation.

NAAS will assess whether your figures meet our technical requirements by comparing each figure against our figure specifications.

Attachment Submitted filename: Peer Review on BK associated with Shallow AC.pdf

# Author comment: Author response to Decision Letter 1

**DOI:** 10.1371/journal.pone.0353272.r002

1 Apr 2026

PONE-D-25-68588

Bullous Keratopathy Associated with a Shallow Anterior Chamber: An Anatomical Risk Phenotype for Corneal Endothelial Decompensation

PLOS One

Journal Requirements:

When submitting your revision, we need you to address these additional requirements.

1.Please ensure that your manuscript meets PLOS ONE's style requirements, including those for file naming. The PLOS ONE style templates can be found at

https://journals.plos.org/plosone/s/file?id=wjVg/PLOSOne_formatting_sample_main_body.pdf and

https://journals.plos.org/plosone/s/file?id=ba62/PLOSOne_formatting_sample_title_authors_affiliations.pdf

Response: Thank you for your comment. We have revised the manuscript to conform to the PLOS ONE style requirements, including the title page, section headings, figure legends, and end-matter formatting. We have also checked the file naming format for the submission files in accordance with the journal guidelines.

2. We note that you have indicated that there are restrictions to data sharing for this study. PLOS only allows data to be available upon request if there are legal or ethical restrictions on sharing data publicly. For more information on unacceptable data access restrictions, please see http://journals.plos.org/plosone/s/data-availability#loc-unacceptable-data-access-restrictions.

Before we proceed with your manuscript, please address the following prompts:

a) If there are ethical or legal restrictions on sharing a de-identified data set, please explain them in detail (e.g., data contain potentially identifying or sensitive patient information, data are owned by a third-party organization, etc.) and who has imposed them (e.g., a Research Ethics Committee or Institutional Review Board, etc.). Please also provide contact information for a data access committee, ethics committee, or other institutional body to which data requests may be sent.

b) If there are no restrictions, please upload the minimal anonymized data set necessary to replicate your study findings to a stable, public repository and provide us with the relevant URLs, DOIs, or accession numbers. For a list of recommended repositories, please see

https://journals.plos.org/plosone/s/recommended-repositories. You also have the option of uploading the data as Supporting Information files, but we would recommend depositing data directly to a data repository if possible.

We will update your Data Availability statement on your behalf to reflect the information you provide.

Response: We thank the editor for this comment. There are no legal or ethical restrictions on sharing the minimal anonymized dataset necessary to replicate the study findings. We have uploaded the dataset as Supporting Information (S1 Dataset). The dataset was reviewed to remove potentially identifying information, including all specific dates, and an English-language version was prepared for submission. The Data Availability Statement in the manuscript reads: “All relevant data are within the manuscript and its Supporting Information files.”

Location: S1 Dataset, manuscript p34 Data availability statement

3. Your ethics statement should only appear in the Methods section of your manuscript. If your ethics statement is written in any section besides the Methods, please delete it from any other section.

Response: We thank the editor for this comment. The ethics statement was already fully described in the Methods section. We therefore removed the duplicate ethics statement from the end of the manuscript and retained it only in the Methods section.

Location: manuscript p5 Study design and ethics

4. Please upload a copy of Figure 3, to which you refer in your text on page 24. If the figure is no longer to be included as part of the submission please remove all reference to it within the text.

Response: We thank the editor for this comment. Figure 3 was included in the revised submission files, and all references to Figure 3 in the main text have been retained accordingly.

Location: Fig 3

5. We note that Figure 2 in your submission contain copyrighted images. All PLOS content is published under the Creative Commons Attribution License (CC BY 4.0), which means that the manuscript, images, and Supporting Information files will be freely available online, and any third party is permitted to access, download, copy, distribute, and use these materials in any way, even commercially, with proper attribution. For more information, see our copyright guidelines: http://journals.plos.org/plosone/s/licenses-and-copyright.

We require you to either (1) present written permission from the copyright holder to publish these figures specifically under the CC BY 4.0 license, or (2) remove the figures from your submission:

1. You may seek permission from the original copyright holder of Figure 2 to publish the content specifically under the CC BY 4.0 license.

We recommend that you contact the original copyright holder with the Content Permission Form (http://journals.plos.org/plosone/s/file?id=7c09/content-permission-form.pdf) and the following text:

“I request permission for the open-access journal PLOS ONE to publish XXX under the Creative Commons Attribution License (CCAL) CC BY 4.0 (http://creativecommons.org/licenses/by/4.0/). Please be aware that this license allows unrestricted use and distribution, even commercially, by third parties. Please reply and provide explicit written permission to publish XXX under a CC BY license and complete the attached form.”

Please upload the completed Content Permission Form or other proof of granted permissions as an "Other" file with your submission.

In the figure caption of the copyrighted figure, please include the following text: “Reprinted from [ref] under a CC BY license, with permission from [name of publisher], original copyright [original copyright year].”

2. If you are unable to obtain permission from the original copyright holder to publish these figures under the CC BY 4.0 license or if the copyright holder’s requirements are incompatible with the CC BY 4.0 license, please either i) remove the figure or ii) supply a replacement figure that complies with the CC BY 4.0 license. Please check copyright information on all replacement figures and update the figure caption with source information. If applicable, please specify in the figure caption text when a figure is similar but not identical to the original image and is therefore for illustrative purposes only.

Response: We thank the editor for this comment. Figure 2 is an original figure created by the authors. Although an earlier version of the manuscript was posted on Research Square, the authors retained the copyright to the preprint and all included figures, while Research Square holds only a non-exclusive license to distribute the content. No copyright transfer to any publisher has occurred. Therefore, the authors retain the right to publish Figure 2 under the CC BY 4.0 license required by PLOS ONE, and no additional third-party permission is required.

6. Thank you for providing your underlying data as Supporting Information.

We note that the data set contains text or data that is not in English. Please note that PLOS is an English-language publisher, so we require data sets to be provided in English as well. Please upload an English-language version of your data set.

This will also allow us to determine if your data follows PLOS standards per our Data Availability policy here: https://journals.plos.org/plosone/s/data-availability.

Response: We thank the editor for this comment. We revised the dataset and uploaded an English-language version as Supporting Information.

Location: S1 Dataset

7. We note that there is identifying data in the Supporting Information file “dataset.pdf”. Due to the inclusion of these potentially identifying data, we have removed this file from your file inventory. Prior to sharing human research participant data, authors should consult with an ethics committee to ensure data are shared in accordance with participant consent and all applicable local laws.

Data sharing should never compromise participant privacy. It is therefore not appropriate to publicly share personally identifiable data on human research participants. The following are examples of data that should not be shared:

-Name, initials, physical address

-Ages more specific than whole numbers

-Internet protocol (IP) address

-Specific dates (birth dates, death dates, examination dates, etc.)

-Contact information such as phone number or email address

-Location data

-ID numbers that seem specific (long numbers, include initials, titled “Hospital ID”) rather than random (small numbers in numerical order)

Data that are not directly identifying may also be inappropriate to share, as in combination they can become identifying. For example, data collected from a small group of participants, vulnerable populations, or private groups should not be shared if they involve indirect identifiers (such as sex, ethnicity, location, etc.) that may risk the identification of study participants.

Additional guidance on preparing raw data for publication can be found in our Data Policy (https://journals.plos.org/plosone/s/data-availability#loc-human-research-participant-data-and-other-sensitive-data) and in the following article: http://www.bmj.com/content/340/bmj.c181.long.

Please remove or anonymize all personal information (<specific identifying information in file to be removed>), ensure that the data shared are in accordance with participant consent, and re-upload a fully anonymized data set. Please note that spreadsheet columns with personal information must be removed and not hidden as all hidden columns will appear in the published file.

Response: We thank the editor for this important comment. We revised the Supporting Information dataset to remove potentially identifying information, including all specific dates, and prepared an English-language version for re-upload. Age is presented only in whole numbers, and no direct personal identifiers are included. We also confirmed that columns containing personal information were removed rather than hidden. The revised dataset has been anonymized in accordance with participant privacy requirements, institutional regulations, and the journal’s data-sharing policy.

Location: S1 Dataset

8. If the reviewer comments include a recommendation to cite specific previously published works, please review and evaluate these publications to determine whether they are relevant and should be cited. There is no requirement to cite these works unless the editor has indicated otherwise.

Response: We thank the editor for this comment. We reviewed the publications recommended by the reviewers, found them relevant to the present study, and incorporated the appropriate citations into the revised Discussion.

Location: manuscript p27-28 Line 340-347, 361-368

9. Please review your reference list to ensure that it is complete and correct. If you have cited papers that have been retracted, please include the rationale for doing so in the manuscript text, or remove these references and replace them with relevant current references. Any changes to the reference list should be mentioned in the rebuttal letter that accompanies your revised manuscript. If you need to cite a retracted article, indicate the article’s retracted status in the References list and also include a citation and full reference for the retraction notice.

Response: We thank the editor for this comment. We carefully reviewed the reference list for completeness and accuracy and revised it as needed in the updated manuscript. We also confirmed that no retracted articles are cited.

Location: manuscript p31-33

Additional Editor Comments:

• Please address particularly the limitations inherent to small sample size.

Response: We thank the editor for this important comment. We revised the Discussion and Limitations sections to more clearly acknowledge the limitations related to the small sample size, particularly in the PAC/Unknown subgroup. Specifically, we emphasized the limited sample size, the relatively short follow-up, the possibility of limited statistical power in some analyses, and the exploratory nature of the findings, and we clarified that larger studies with longer follow-up are needed.

Location: manuscript p30 Limitations

[Note: HTML markup is below. Please do not edit.]

Reviewers' comments:

Reviewer's Responses to Questions

Comments to the Author

5. Review Comments to the Author

Please use the space provided to explain your answers to the questions above. You may also include additional comments for the author, including concerns about dual publication, research ethics, or publication ethics. (Please upload your review as an attachment if it exceeds 20,000 characters)

Reviewer #1: The work highlights an important and often under-recognized phenotype, and the imaging documentation is excellent. To further strengthen the study and align it with current understanding of postoperative endothelial and stromal behavior, I would encourage you to address the following points.

-Please discuss the recent literature that it has demonstrated that PSR are not merely descriptive findings but structural biomarkers of stromal overhydration and lamellar micro-distortion. In particular, preoperative PSR correlate with slower visual recovery and postoperative PSR strongly predict rebubbling and graft instability (Ventura et al., Cornea 2024). Including this discussion will contextualize your observations and provide a more mechanistic interpretation of stromal morphology in your cohort.

Response: Thank you for this insightful comment. We have expanded the Discussion to incorporate the recent literature on posterior stromal ripples (PSR). Specifically, we clarified that PSR are not merely descriptive findings on anterior segment optical coherence tomography, but structural biomarkers of stromal overhydration and lamellar micro-distortion after DMEK. We also added discussion of the reported associations between preoperative PSR and slower visual recovery, as well as between postoperative PSR and rebubbling/graft instability. In addition, we related these findings to the posterior stromal irregularity observed in our illustrative case, while acknowledging that PSR were not systematically assessed in the present cohort.

Location: manuscript p27-28 Line 340-347, 361-368

-Discuss the role of subclinical inflammation. AS-OCT–based studies have shown that subclinical anterior chamber inflammation is almost universally present after DMEK, and higher postoperative inflammatory load (measured by ARI index and hyperreflective dots) is linked to the development of postoperative PSR and rebubbling (Vaccaro et al., Ophthalmology Science 2026). Incorporating this concept can help explain why some eyes demonstrate unexpected endothelial stress or postoperative behavior despite uneventful surgery.

Response: Thank you for this important comment. We have expanded the Discussion to address the role of subclinical postoperative inflammation after DMEK. Specifically, we added discussion of AS-OCT–based evidence showing that higher postoperative inflammatory load, reflected by the ARI index and hyperreflective dots, is associated with PSR and with postoperative rebubbling/graft instability. We also noted that this concept may help explain variable postoperative behavior despite apparently uneventful surgery.

Location: manuscript p27-28 Line 340-347, 361-368

-clarify the mechanistic link between anatomy and endothelial vulnerability. Your hypothesis of a shallow-anterior-chamber risk phenotype is intriguing, but discussing how crowding, micro-shear forces, and low-grade inflammation may contribute to chronic endothelial stress would strengthen the biological plausibility of the proposed mechanism.

Response: Thank you for this helpful suggestion. We have expanded the Discussion to clarify the potential mechanistic link between shallow anterior chamber anatomy and endothelial vulnerability. Specifically, we added discussion of anterior segment crowding, altered aqueous flow and micro-shear forces, intermittent IOP elevation, episodic iridocorneal contact, and low-grade inflammation as possible c

Attachment Submitted filename: point-to-point.docx

# Editor report: Decision Letter 1

**DOI:** 10.1371/journal.pone.0353272.r003

**Authors:** Vaitheeswaran Ganesan Lalgudi

25 May 2026

PONE-D-25-68588R1

Bullous keratopathy associated with a shallow anterior chamber: an anatomical risk phenotype for corneal endothelial decompensation

PLOS One

Dear Dr. Hayashi,

Thank you for submitting your manuscript to PLOS ONE. After careful consideration, we feel that it has merit but does not fully meet PLOS ONE’s publication criteria as it currently stands. Therefore, we invite you to submit a revised version of the manuscript that addresses the points raised during the review process.

==============================

**ACADEMIC EDITOR: Please read comments mentioned by editor**

Please submit your revised manuscript by Jul 09 2026 11:59PM. If you will need more time than this to complete your revisions, please reply to this message or contact the journal office at plosone@plos.org. When you're ready to submit your revision, log on to https://www.editorialmanager.com/pone/ and select the 'Submissions Needing Revision' folder to locate your manuscript file.

Please include the following items when submitting your revised manuscript:

- A letter that responds to each point raised by the academic editor and reviewer(s). You should upload this letter as a separate file labeled 'Response to Reviewers'.
- A marked-up copy of your manuscript that highlights changes made to the original version. You should upload this as a separate file labeled 'Revised Manuscript with Track Changes'.
- An unmarked version of your revised paper without tracked changes. You should upload this as a separate file labeled 'Manuscript'.

If you would like to make changes to your financial disclosure, please include your updated statement in your cover letter. Guidelines for resubmitting your figure files are available below the reviewer comments at the end of this letter.

If applicable, we recommend that you deposit your laboratory protocols in protocols.io to enhance the reproducibility of your results. Protocols.io assigns your protocol its own identifier (DOI) so that it can be cited independently in the future. For instructions see: https://journals.plos.org/plosone/s/submission-guidelines#loc-laboratory-protocols. Additionally, PLOS ONE offers an option for publishing peer-reviewed Lab Protocol articles, which describe protocols hosted on protocols.io. Read more information on sharing protocols at https://plos.org/protocols?utm_medium=editorial-email&utm_source=authorletters&utm_campaign=protocols.

As the corresponding author, your ORCID iD is verified in the submission system and will appear in the published article. PLOS supports the use of ORCID, and we encourage all coauthors to register for an ORCID iD and use it as well. Please encourage your coauthors to verify their ORCID iD within the submission system before final acceptance, as unverified ORCID iDs will not appear in the published article. *Only*  the individual author can complete the verification step; PLOS staff *cannot*  verify ORCID iDs on behalf of authors.

We look forward to receiving your revised manuscript.

Kind regards,

Vaitheeswaran G Lalgudi, M.D.

Academic Editor

PLOS One

Journal Requirements:

If the reviewer comments include a recommendation to cite specific previously published works, please review and evaluate these publications to determine whether they are relevant and should be cited. There is no requirement to cite these works unless the editor has indicated otherwise.

Please review your reference list to ensure that it is complete and correct. If you have cited papers that have been retracted, please include the rationale for doing so in the manuscript text, or remove these references and replace them with relevant current references. Any changes to the reference list should be mentioned in the rebuttal letter that accompanies your revised manuscript. If you need to cite a retracted article, indicate the article’s retracted status in the References list and also include a citation and full reference for the retraction notice.

Additional Editor Comments:

While this is an interesting paper as previously mentioned by the reviewers, there are several concerns that need to be addressed to improve the quality and validity of this article.

1. Firstly, to improve the repeatability of the methodology, we need to have a clear explanation on how PAC was diagnosed. Was it just based on ACD, or based on gonioscopic findings as well? There seems to be no description of that. If gonioscopy was performed, was there any variation on the grade of the angle, including presence or absence and extent of PAS in the patients studied? Was ASOCT performed to analyze the trabecular iris angle (TIA)? And, please describe 'dark spots' on specular microscopy in the methodology.

2. As cornea specialists, when we see patients with BK without evidence of FECD (clinically/on specular of affected/fellow eye), the following are the differentials. Number 1 is cataract surgery or phaco energy induced endothelial loss or PBK. Especially in eyes with shallow ACD or with denser cataracts, the impact of phaco energy on the endothelium is significant. IOL position is a significant concern as well. Sulcus IOLs or UGH concerns or ACIOLs are known to increase the risk of corneal decompensation. Other aspects to rule out are a viral masquerade. Patients with a HSV or CMV endotheliitis in the past can also present with endothelial dysfunction and can improve with oral antivirals and topical steroids. While trying to describe a group of BK eyes with PAC without APAC or ALI, one of the major concerns is the fact that the authors seem to be completely ignoring the role and impact of cataract surgery and other above mentioned factors in these patients. The following parameters need to be added to the article. a) What was the timeline of the cataract surgery before presenting with corneal edema? b) Cataract surgery parameters including CDE (cumulative dissipated energy), IOL used and position of the IOL, c) How long prior to Cataract surgery was LPI performed? d) Pre-cataract surgery ECD on specular microscopy. e) Was any aqueous tap performed for ruling out HSV/CMV IgG/IgM PCR in BK patients with no evidence of FECD?

3. In the results section, what does 'baseline' visit represent? Is it prior to cataract surgery or prior to DMEK surgery?

4. Table 1 is confusing. After the row on 'Breakdown of BK', please create a separate table with new headers to make it easy for readers.

5. In table 1, 19 patients are mentioned to have Laser iridotomy. However, your PAC group with ALI is only 18. Why is this discrepancy?

6. Line 315 mentions that PEX had deeper chambers than those with PAC eyes. However, Table 2 mentions PEX ACD mean as 2.09mm versus PAC group with 2.20 mm mean. Please fix this discrepancy.

7. The following article has also previously described CECD cell loss in PACS eyes without ALI or angle closure attacks. Kusumi Y, Yamamoto M, Fukui M, Yamada M. Morphological Features in Eyes with Prominent Corneal Endothelial Cell Loss Associated with Primary Angle-Closure Disease. J Clin Med. 2025 Jul 29;14(15):5364. doi: 10.3390/jcm14155364. PMID: 40806986; PMCID: PMC12346976. This article points out the role of keratometry or radius of curvature in CECD loss in PACS eyes. Are you able to include corneal curvature in your patients in the study as well? Please also include this study in the discussion section.

[Note: HTML markup is below. Please do not edit.]

[NOTE: If reviewer comments were submitted as an attachment file, they will be attached to this email and accessible via the submission site. Please log into your account, locate the manuscript record, and check for the action link "View Attachments". If this link does not appear, there are no attachment files.]

To ensure your figures meet our technical requirements, please review our figure guidelines: https://journals.plos.org/plosone/s/figures

You may also use PLOS’s free figure tool, NAAS, to help you prepare publication quality figures: https://journals.plos.org/plosone/s/figures#loc-tools-for-figure-preparation.

NAAS will assess whether your figures meet our technical requirements by comparing each figure against our figure specifications.

# Author comment: Author response to Decision Letter 2

**DOI:** 10.1371/journal.pone.0353272.r004

13 Jun 2026

Response to Academic Editor

Manuscript ID: PONE-D-25-68588R1

Title: Bullous keratopathy associated with a shallow anterior chamber: an anatomical risk phenotype for corneal endothelial decompensation

Dear Dr. Lalgudi,

We sincerely thank you and the reviewers for the careful evaluation of our manuscript and for the constructive comments. We have revised the manuscript accordingly to improve the clarity, reproducibility, and validity of the study. All changes have been incorporated into the revised manuscript and are indicated in the tracked-changes version.

Below, we provide a point-by-point response to each comment.

Comment 1

Editor’s comment:

Firstly, to improve the repeatability of the methodology, we need to have a clear explanation on how PAC was diagnosed. Was it just based on ACD, or based on gonioscopic findings as well? There seems to be no description of that. If gonioscopy was performed, was there any variation on the grade of the angle, including presence or absence and extent of PAS in the patients studied? Was ASOCT performed to analyze the trabecular iris angle (TIA)? And, please describe 'dark spots' on specular microscopy in the methodology.

Response:

Thank you for this important comment. We agree that the previous terminology may have been misleading. In the original manuscript, we used the terms “PAC/ALI” and “PAC/Unknown.” However, because the former “PAC/Unknown” group did not have sufficient documentation to support a strict diagnosis of PACD based on uniform gonioscopic criteria, we revised the terminology throughout the manuscript. Specifically, we renamed this group as the “shallow anterior chamber (shallow-AC) group” and clarified that this classification was intended to represent a shallow anterior chamber phenotype rather than a definitive diagnosis of PACD.

We also revised the Methods section to clarify how PACD was diagnosed. The PACD/ALI group was defined based on medical records documenting a history of argon laser iridotomy performed for a clinically diagnosed PACD-spectrum condition, supported when available by gonioscopic angle closure and/or a clinical diagnosis by glaucoma or corneal specialists. We further clarified that, because of the retrospective nature of the study, standardized gonioscopic grading and the extent of peripheral anterior synechiae were not consistently available and were therefore not included as analytic variables.

In addition, we clarified that AS-OCT was used to measure ACD and CCT, but angle parameters, including the trabecular iris angle, were not uniformly measured and were not used for group classification or statistical analyses. Finally, we added a definition of dark spots on specular microscopy and clarified that dark spots alone were not used as independent diagnostic criteria for FECD.

Location:

Abstract; Methods, Patients and data collection; Methods, Ophthalmic examinations; Results; Discussion; Figure legends; Supporting Information.

Comment 2

Editor’s comment:

As cornea specialists, when we see patients with BK without evidence of FECD (clinically/on specular of affected/fellow eye), the following are the differentials. Number 1 is cataract surgery or phaco energy induced endothelial loss or PBK. Especially in eyes with shallow ACD or with denser cataracts, the impact of phaco energy on the endothelium is significant. IOL position is a significant concern as well. Sulcus IOLs or UGH concerns or ACIOLs are known to increase the risk of corneal decompensation. Other aspects to rule out are a viral masquerade. Patients with a HSV or CMV endotheliitis in the past can also present with endothelial dysfunction and can improve with oral antivirals and topical steroids. While trying to describe a group of BK eyes with PAC without APAC or ALI, one of the major concerns is the fact that the authors seem to be completely ignoring the role and impact of cataract surgery and other above mentioned factors in these patients. The following parameters need to be added to the article. a) What was the timeline of the cataract surgery before presenting with corneal edema? b) Cataract surgery parameters including CDE (cumulative dissipated energy), IOL used and position of the IOL, c) How long prior to Cataract surgery was LPI performed? d) Pre-cataract surgery ECD on specular microscopy. e) Was any aqueous tap performed for ruling out HSV/CMV IgG/IgM PCR in BK patients with no evidence of FECD?

Response:

We thank the Editor for this important comment. We agree that cataract surgery-related endothelial injury, IOL-related factors, and viral endotheliitis are important differential considerations when evaluating bullous keratopathy without evidence of FECD. To address this concern, we reviewed additional cataract surgery-related variables in the shallow anterior chamber phenotype group and added these data to the Results section under “Patient characteristics” and to S2 Table.

Specifically, we added information on the timing of cataract surgery relative to corneal edema confirmation, cumulative dissipated energy, IOL type and position, the interval from ALI to cataract surgery, pre-cataract surgery ECD, and clinical findings and aqueous humor testing related to possible viral endotheliitis. Among the 28 eyes in the shallow anterior chamber phenotype group, 22 eyes underwent cataract surgery as part of a planned staged surgical approach before DMEK after corneal endothelial decompensation or BK had already been recognized. Cumulative dissipated energy was not available in the medical records. Among eyes with available IOL position data, all IOLs were placed in the bag, and no sulcus IOL or anterior chamber IOL was documented. The interval from ALI to cataract surgery and pre-cataract surgery ECD are summarized in S2 Table. Clinical findings suggestive of viral endotheliitis were noted in one eye, and aqueous humor testing, when performed, was negative.

These additions clarify that cataract surgery-related factors, IOL position, prior ALI, pre-cataract endothelial status, and possible viral masquerade were specifically reviewed in response to the Editor’s concern.

Although cumulative dissipated energy was not available because of the retrospective nature of the study, the newly added data indicate that most cataract surgeries in this subgroup were performed as planned staged procedures after endothelial decompensation or BK had already been recognized, and that no sulcus IOL or anterior chamber IOL was documented among evaluable eyes.

Location:

Results, Patient characteristics; Supporting Information, S2 Table.

Comment 3

Editor’s comment:

In the results section, what does 'baseline' visit represent? Is it prior to cataract surgery or prior to DMEK surgery?

Response:

Thank you for this important comment. To avoid ambiguity, we revised the Methods section and removed the term “baseline” from the relevant descriptions. We now specify that VA, CCT, IOP, the number of IOP-lowering medications, axial length, and preoperative donor ECD were recorded before DMEK. ACD was evaluated separately as pre-cataract surgery ACD and post-cataract surgery ACD. We also clarified that post-cataract surgery ACD was measured at the pre-DMEK assessment.

Location: Methods, Ophthalmic examinations; Results, Patient characteristics; Supporting Information, S4 and S5 Tables.

Comment 4

Editor’s comment:

Table 1 is confusing. After the row on 'Breakdown of BK', please create a separate table with new headers to make it easy for readers.

Response:

Thank you for this helpful suggestion. We revised Table 1 to improve readability. Specifically, we separated the original “Breakdown of BK” section from the main patient demographics and clinical characteristics table and created a separate table with new headers for BK cases. The main patient characteristics are now presented in Table 1A, and the breakdown of BK cases is presented separately in Table 1B. We also revised the corresponding text citation in the Results section.

Location: Results, Patient characteristics; Tables 1A and 1B.

Comment 5

Editor’s comment:

In table 1, 19 patients are mentioned to have Laser iridotomy. However, your PAC group with ALI is only 18. Why is this discrepancy?

Response:

Thank you for pointing this out. This discrepancy was due to a transcription error in the previous version of Table 1. We reviewed the dataset and confirmed that 18 eyes had a history of laser iridotomy, which is consistent with the number of eyes in the PACD/ALI group. We corrected the value in Table 1A accordingly.

Location: Table 1A.

Comment 6

Editor’s comment:

Line 315 mentions that PEX had deeper chambers than those with PAC eyes. However, Table 2 mentions PEX ACD mean as 2.09mm versus PAC group with 2.20 mm mean. Please fix this discrepancy.

Response:

We thank the editor for pointing out this discrepancy. We agree that the original statement was imprecise. In Table 2, the mean pre-cataract surgery ACD was 2.09 mm in the PEX subgroup and 2.20 mm in the shallow-AC subgroup; therefore, it was inappropriate to state that PEX was associated with deeper chambers in this context. We deleted the sentence “Conversely, FECD and PEX were associated with deeper chambers” and revised the Discussion to avoid this misleading interpretation. The revised sentence now reads: “Together, these findings suggest that eyes with the shallow anterior chamber phenotype may have anterior segment characteristics that differ from those of eyes with FECD.”

Location: Discussion, first paragraph.

Comment 7

Editor’s comment:

The following article has also previously described CECD cell loss in PACS eyes without ALI or angle closure attacks. Kusumi Y, Yamamoto M, Fukui M, Yamada M. Morphological Features in Eyes with Prominent Corneal Endothelial Cell Loss Associated with Primary Angle-Closure Disease. J Clin Med. 2025 Jul 29;14(15):5364. doi: 10.3390/jcm14155364. PMID: 40806986; PMCID: PMC12346976. This article points out the role of keratometry or radius of curvature in CECD loss in PACS eyes. Are you able to include corneal curvature in your patients in the study as well? Please also include this study in the discussion section.

Response:

We appreciate the Editor’s important suggestion. We agree that the study by Kusumi et al. is highly relevant, because it reported prominent corneal endothelial cell density loss in eyes with primary angle-closure disease, including primary angle-closure suspect eyes without a history of acute angle-closure attacks or laser iridotomy, and suggested a potential role of corneal curvature radius in endothelial vulnerability. We have incorporated this study into the Discussion.

Unfortunately, keratometric data and corneal curvature parameters were not consistently available because of the retrospective nature of this study and the long study period. Therefore, we were unable to perform a reliable corneal curvature analysis in the present cohort.

Location: Discussion.

Additional revisions

In addition to the changes made in response to the editor’s comments, we carefully reviewed the manuscript for clarity, consistency, and typographical errors.

Sincerely,

Takahiko Hayashi

On behalf of all authors

Attachment Submitted filename: Point_to_point_DMEK_for_shallow_AC.docx

# Editor report: Decision Letter 2

**DOI:** 10.1371/journal.pone.0353272.r005

**Authors:** Vaitheeswaran Ganesan Lalgudi

22 Jun 2026

Bullous keratopathy associated with a shallow anterior chamber: an anatomical risk phenotype for corneal endothelial decompensation

PONE-D-25-68588R2

Dear Dr. Hayashi,

We’re pleased to inform you that your manuscript has been judged scientifically suitable for publication and will be formally accepted for publication once it meets all outstanding technical requirements.

Within one week, you’ll receive an e-mail detailing the required amendments. When these have been addressed, you’ll receive a formal acceptance letter and your manuscript will be scheduled for publication.

An invoice will be generated when your article is formally accepted. Please note, if your institution has a publishing partnership with PLOS and your article meets the relevant criteria, all or part of your publication costs will be covered. Please make sure your user information is up-to-date by logging into Editorial Manager at Editorial Manager® and clicking the ‘Update My Information' link at the top of the page. For questions related to billing, please contact billing support.

If your institution or institutions have a press office, please notify them about your upcoming paper to help maximize its impact. If they’ll be preparing press materials, please inform our press team as soon as possible -- no later than 48 hours after receiving the formal acceptance. Your manuscript will remain under strict press embargo until 2 pm Eastern Time on the date of publication. For more information, please contact onepress@plos.org.

Kind regards,

Vaitheeswaran G Lalgudi, M.D.

Academic Editor

PLOS One

Additional Editor Comments (optional):

All the comments have been addressed appropriately. I congratulate the team for their work.

Reviewers' comments:

# Editor report: Acceptance letter

**DOI:** 10.1371/journal.pone.0353272.r006

**Authors:** Vaitheeswaran Ganesan Lalgudi

PONE-D-25-68588R2

PLOS One

Dear Dr. Hayashi,

I'm pleased to inform you that your manuscript has been deemed suitable for publication in PLOS One. Congratulations! Your manuscript is now being handed over to our production team.

At this stage, our production department will prepare your paper for publication. This includes ensuring the following:

* All references, tables, and figures are properly cited

* All relevant supporting information is included in the manuscript submission,

* There are no issues that prevent the paper from being properly typeset

You will receive further instructions from the production team, including instructions on how to review your proof when it is ready. Please keep in mind that we are working through a large volume of accepted articles, so please give us a few days to review your paper and let you know the next and final steps.

Lastly, if your institution or institutions have a press office, please let them know about your upcoming paper now to help maximize its impact. If they'll be preparing press materials, please inform our press team within the next 48 hours. Your manuscript will remain under strict press embargo until 2 pm Eastern Time on the date of publication. For more information, please contact onepress@plos.org.

You will receive an invoice from PLOS for your publication fee after your manuscript has reached the completed accept phase. If you receive an email requesting payment before acceptance or for any other service, this may be a phishing scheme. Learn how to identify phishing emails and protect your accounts at https://explore.plos.org/phishing.

If we can help with anything else, please email us at customercare@plos.org.

Thank you for submitting your work to PLOS ONE and supporting open access.

Kind regards,

PLOS ONE Editorial Office Staff

on behalf of

Dr. Vaitheeswaran Ganesan Lalgudi

Academic Editor

PLOS One
